# Non-invasive mapping of the temporal processing hierarchy in the human visual cortex

**DOI:** 10.1371/journal.pcbi.1014434

**Published:** 2026-07-10

**Authors:** Katharina Eickhoff, Arjan Hillebrand, Tomas Knapen, Maartje C. de Jong, Serge O. Dumoulin

**Affiliations:** 1 Spinoza Centre for Neuroimaging, Amsterdam, the Netherlands; 2 Computational Cognitive Neuroscience and Neuroimaging, Netherlands Institute for Neuroscience, Amsterdam, the Netherlands; 3 Experimental and Applied Psychology, Vrije Universiteit, Amsterdam, the Netherlands; 4 Clinical Neurophysiology and Magnetoencephalography Centre, Amsterdam UMC, Amsterdam, the Netherlands; 5 Amsterdam Neuroscience, Brain Imaging, Amsterdam, the Netherlands; 6 Amsterdam Neuroscience, Systems and Network Neuroscience, Amsterdam, the Netherlands; 7 Experimental Psychology, Utrecht University, Utrecht, the Netherlands; ShanghaiTech University, CHINA

## Abstract

Vision, and brain processing more broadly, is inherently dynamic across space and time, so understanding brain function requires consideration of both spatial and temporal dimensions. However, simultaneously capturing the fine spatial details and the rapid temporal dynamics of visual processing remains a major challenge, resulting in a gap in our understanding of spatiotemporal dynamics. Here, we introduce a forward modeling technique that bridges high-spatial resolution fMRI with high-temporal resolution MEG, enabling us to non-invasively estimate different levels of the visual hierarchy in humans and their involvement in visual processing with millisecond precision. Using fMRI, levels of the visual hierarchy were identified by measuring individuals’ population receptive fields and determining visual field maps. We predicted how much the activity patterns in each visual field map would contribute to brain responses measured with MEG. By comparing these predicted responses with the measured MEG responses, we assessed how much a given visual field map contributed to the measured MEG response, and, most importantly, when. We combined information from all MEG sensors and revealed a cortical processing hierarchy across visual field maps. We validated the method using cross-validations and demonstrated that the model generalized across MEG sensor types, stimulus shapes, and was robust to the number of visual field maps included in the model. The primary visual cortex captured most of the variance in the MEG sensors and did so earlier in time than extrastriate regions. We effectively combined the advantages of two very different neuroimaging techniques, opening avenues for answering research questions that require recordings with high spatiotemporal detail. By bridging traditionally separate areas of research, our approach helps close longstanding gaps in our understanding of brain function.

## Author summary

Vision doesn’t happen instantaneously, but unfolds over time. While we understand a lot about how the brain processes visual space, understanding how the brain processes information over both space and time is much more challenging. Vision happens incredibly fast, space and time are tightly linked, but current technology is limited in capturing these spatiotemporal dynamics. Here, we developed a method that combines two non-invasive human brain imaging techniques using computational models: fMRI, which provides high spatial detail, and MEG, which captures millisecond-level timing. We use fMRI to model the detailed layout of the visual system, and predict how this spatial layout responds to specific visual stimuli and compare these predictions with MEG recordings. This allows us to pinpoint not just where visual processing happens, but also *when*. Our results reveal a clear processing hierarchy: primary visual cortex responds first and explains most of the MEG signal, while higher-level visual areas activate later. We show that our method works across different stimuli, MEG sensor types, and brain areas, proving its robustness. This approach offers a new way to track how information flows through the living human brain, opening up new possibilities to study vision in both health and disease.

## Introduction

Vision is not instantaneous but evolves over time [1–3]. To rapidly interpret the world, the visual system relies on highly interconnected neuronal populations that form visual field maps that exchange information within hundreds of milliseconds [4–8]. This communication is dynamic, adapting to factors such as stimulus features, the current state of the visual system, and contextual influences, such as current task-demands [9–11]. Invasive recordings, mostly conducted in animal models, played a crucial role in uncovering the neural basis of visual processing. However, human visual processing may differ from animals in terms of visual system layout and differences in the amount of cognitive modulations from higher order regions of the brain [12].

To understand visual perception, we need to understand how information flows through the human brain. While invasive studies suggest a rough visual processing hierarchy, pinpointing the precise timing of neuronal responses across the visual cortex remains challenging in the healthy human brain. The challenge lies in the extraordinarily fast visual processing, the tight link between space and time, and the difficulty of measuring with high spatial and temporal resolution non-invasively in the living human brain.

Bridging the gap between spatial and temporal precision is essential to understand how visual information flows through the human brain. Functional magnetic resonance imaging (fMRI) provides high spatial resolution but lacks millisecond-scale timing. Magnetoencephalography (MEG) and other neurophysiological methods offer superior temporal resolution but struggle to pinpoint the exact cortical sources at millimeter resolution [3,13,14].

Here, we advanced pRF modeling approaches to estimate not only the contribution of individual visual field maps along the visual hierarchy, but also *when* they do so with millisecond precision. Building on our previous work that combines pRF models with fMRI and MEG [15,16], we now integrate signals across all MEG sensors to capture temporal information in specific cortical regions. Importantly, this approach allows us to characterize neural processing within brain regions using combined sensor data, rather than limiting our analysis to the sensor level. Biologically-inspired models, such as the pRF model, are valuable because they offer interpretable, mechanistic insights into how the brain processes information, linking neural activity to specific stimulus features, whereas methods like representational similarity analysis (RSA) focus primarily on similarities in activation patterns without tying them directly to underlying neural computations or properties.

By using fMRI to predict pRFs at the cortical level, applying a forward model to project these into MEG measurement space, and evaluating response latencies, we provide detailed temporal activation windows of visual processing across the visual hierarchy. Specifically, primary visual cortex (V1) responded fastest, while extrastriate areas followed later on. Last, to demonstrate that the pRF model captures local neuronal information processing, we show that the pRF model generalized over MEG sensor types and stimulus shapes, and was robust to the number of regressors used in the model, highlighting that our model captures neural information processing, and showing the versatility and reliability of the method. Our modeling approach opens up new avenues to understand the differences in timing along the visual hierarchy under various circumstances, task-demands and clinical conditions.

## Results

**Fig 1:**
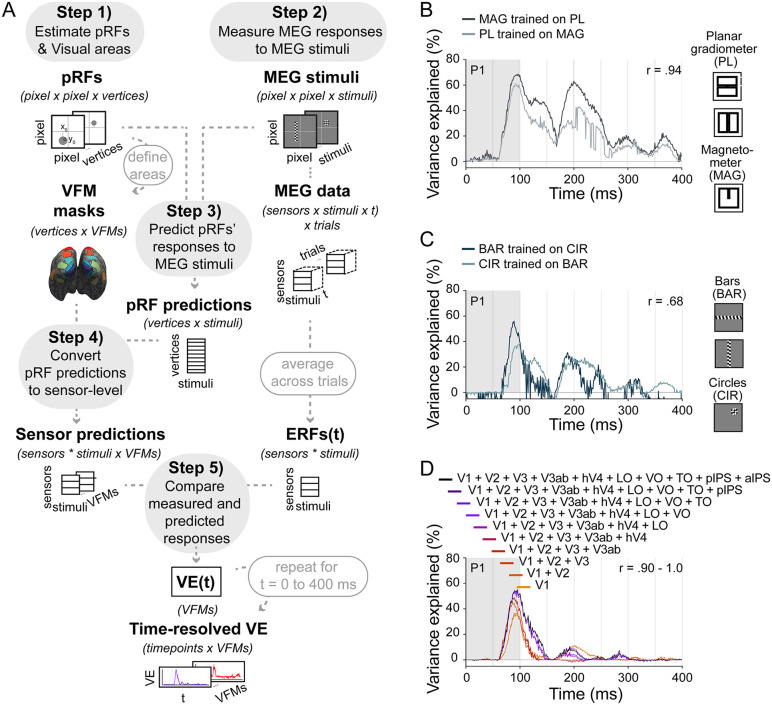
Overview modeling pipeline and model validation results. **A. Modeling pipeline**: *Step 1)* We estimated each participant’s population receptive fields (pRFs) and defined ten visual field maps and clusters (VFMs) along the cortical surface. *Step 2)* MEG responses to bar and circle stimuli were recorded and averaged to event-related fields (ERFs). *Step 3)* pRF-based predictions were computed by matrix multiplying stimulus apertures with the pRFs. *Step 4)* The predictions were projected to MEG sensor space using a gain matrix. *Step 5)* A cross-validated ridge regression assessed how well VFM-based predictions explained the measured MEG response (variance explained; VE) at a given timepoint (t). **B**. **Sensor-type generalization**: Full model’s cross-validated variance explained for the model trained on one sensor type and tested on the left-out sensor type’s data for participant 1 (S1A Fig for all participants). The two time-courses were highly similar indicating our model generalized across sensor types (Pearson correlation coefficient: r(398)=0.94, p < 0.001). The gray shaded area indicates the period when the stimulus was shown. **C**. **Stimulus-type generalization**: Cross-validated variance explained for the model trained on one stimulus type and tested on the left-out stimulus type’s data (bars vs circles) for participant 1 (S1B Fig for all participants). Again the two time-courses were highly similar indicating that our model generalized across stimulus types (r(398) = 0.68, p < 0.001). **D**. **Robustness to number visual field maps and clusters**: We estimated V1’s cross-validated variance explained time-course including different numbers of extrastriate VFMs for participant 1 (S1C Fig for all participants); i.e., ranging from including all ten VFMs (black line), to only including V1 (yellow line). The time-courses were highly similar, indicating that V1’s time-course estimates were robust to the number of extrastriate visual field maps and clusters included in the model (minimum and maximum r(398) = 0.90 and 1.0, p < 0.001).

To estimate the time-courses of visual field maps along the visual hierarchy, we build a forward modeling approach, using population receptive field (pRF) models, to link high-spatial resolution fMRI and millisecond resolution MEG. First, we estimated population receptive fields using fMRI (Fig 1A Step 1; [17]) and defined visual field maps and clusters (VFMs) [18–21]. Second, we collected MEG responses to contrast-defined bar and circle shapes (Fig 1A Step 2). Third, we used the pRF models to predict responses to the same stimuli, such that activity of a given vertex scaled with the degree of overlap between the vertex’ pRF model and the stimulus (Fig 1A Step 3). Next, we converted the pRF predictions to the MEG sensor-space, by applying masked gain matrices to the predictions that convert the VFMs’ cortical responses to the sensor level (Fig 1A Step 4; [22,23]). The VFMs’ sensor predictions were then compared to the measured responses in a cross-validated ridge regression [24], determining the contribution of visual field maps and clusters with millisecond resolution (Fig 1A Step 5).

### Model generalizes across sensors, stimuli and number of visual field maps and clusters

To assess the generalizability of our model, we trained the model on one of the sensor types, and tested the model fit on the left-out sensor type in all combinations, i.e., magnetometers versus planar gradiometers. The cross-validated estimated time-courses were highly correlated with the original time-courses, indicating high generalizability over sensor types (r(398) =  .94, p < .001; r(398) =  .96, p < .001; r(398) =  .96, p < .001; r(398) =  .93, p < .001; and r(398) =  .98, p < .001 for participant 1–5, respectively; Figs 1B and S1A for the other four participants).

In addition, in a similar procedure, we tested the generalizability of the models across stimulus shapes, i.e., bars vs circles. We used one stimulus type for training the model, and evaluated the model on the left-out stimulus type. The generalizability of the model was high across participants (r(398) =  .68, p < .001; r(398) =  .31, p < .001; r(398) =  .32, p < .001; r(398) =  .31, p < .001; and r(398) =  .52, p < .001 for participants 1–5, respectively; Fig 1C for participant 1; S1B Fig for the other four participants).

We identified four visual field maps and six visual field map clusters, but we could have selected more or fewer. To assess the influence of the number of included visual field maps on V1’s estimated time-course, we calculated the fit using only V1 to explain the data, and then incrementally added all visual field maps and clusters up to including all ten areas in the model. We found high Pearson correlation coefficients between V1’s variance explained time-course that was obtained when only V1 was used in the model as compared to when the other visual field maps or clusters were included in the model. The minimum and maximum (i.e., the range) for each participant, was r(398) =  .90 – 1.0, p < .001; r(398) =  .91 -  .98, p < .001; r(398) =  .83 -  .98, p < .001; r(398) =  .97 -  .99, p < .001; and r(398) =  .99 – 1.0, p < .001 for participants 1–5, respectively, showing that V1’s fits across models with different numbers of predictors were stable for all participants (Fig 1D for participant 1; S1C Fig for the other four participants).

We also examined whether exclusion of V1 would alter the remaining visual field maps and clusters’ fits. We found that most visual field maps and clusters were stable when removing V1 from the model fit. For some visual field maps or clusters, namely those that had predictions that correlated strongly with V1’s predicted values, we observed changes in the variance explained time-course (S2 Fig).

Together, the results suggest that our method is both generalizable and robust, allowing detailed investigation into the contribution of individual visual field maps and clusters in explaining visual responses, as well as their timing in doing so.

### V1 explains most variance in measured MEG responses

**Fig 2:**
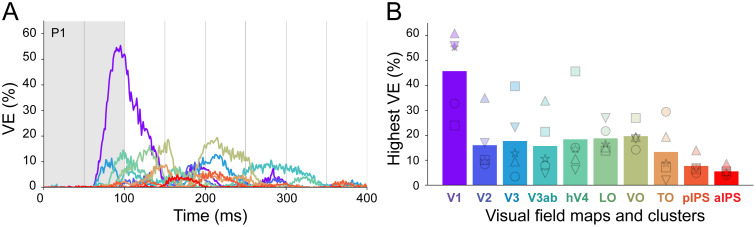
Visual field maps and clusters’ variance explained time-courses and maximal amplitude. **A**. Participant 1’s cross-validated variance explained (VE) time-courses for each visual field map and cluster from 0 to 400 ms after stimulus onset (color coded as in B; for other participants see S3A Fig). The gray shaded area indicates the period when the stimulus was shown. **B**. Visual field maps and cluster’s highest variance explained during the 400 ms time window. Bars indicate the mean over participants; individual markers each participant (star, circle, square, downward triangle, upward triangle for participant 1 to 5).

We calculated how much variance each visual field map and cluster explained in the measured signal (Fig 1A Step 5). We found that V1 reached the highest variance explained across most participants (Fig 2).

### Distinctive processing latencies across visual field maps and clusters

To quantify the timing of individual visual field maps and clusters, we identified the activity window in which they were contributing to the measured signal, by calculating when they reached 25, 50 and 75% of the normalized cumulative curve of the variance explained time-course (S3B Fig). This method avoids biases in setting thresholds and other parameters necessary for methods such as peak detection (S4 Fig).

**Fig 3:**
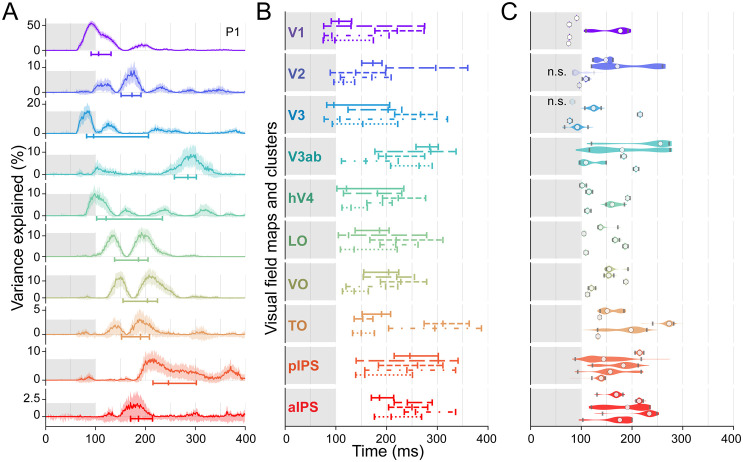
Variance explained time-courses and activation timing for different visual field maps and clusters. **A.** Median (solid) and 95% confidence intervals (shaded) for cross-validated variance explained for different visual field maps and clusters (rows) for participant 1 for 0 to 400 ms after stimulus onset (similar data as Fig 2A but with change of scale, see other participants in S3A Fig). The gray shaded area indicates stimulus presentation. The vertical ticks on the horizontal line below each variance explained time-course mark the time at which normalized cumulative variance explained reached 25, 50, and 75% of the total across the shown 400-ms time window. Note that the y-axes differ for each visual field map and cluster. **B**. 25, 50, 75% of the normalized cumulative variance explained time-course (S3B Fig), for all five participants (P1: solid line, P2: long dashed line, P3: short dashed line, P4: dash-dotted line, P5: dotted line). **C**. Latency of the 25% point. Distributions are shown as violin plots with the 95% confidence intervals. Significant differences between V1 and extrastriate latencies are indicated in opaque shading (Bonferroni-corrected p < 0.05), while non-significant differences are shown by transparent shading and marked ‘n.s.’.

We found a trend of increasing latency along the visual hierarchy (from V1 to aIPS) across participants (Fig 3). Across participants, the average timing of the 25th and 75th percentile of the cumulative VE time-course was 89 and 199 ms for V1, respectively, and 151 and 258 ms for the extrastriate regions.

To quantify the increasing latency from V1 to extrastriate cortex, we compared the latencies of the 25% points between V1 and extrastriate areas using a Fisher permutation test (10,000 permutations). These distributions are visualized as violin plots, reflecting the spread of latencies across 120 cross-validation folds. The white dot indicates the median onset across the 120 folds, and gray horizontal lines denote the 95% confidence interval of the latency distribution derived from these folds. We found that almost every extrastriate region in every participant had a significantly later latency than V1, except for V2 in P3 and V3 in P1 (Bonferroni-corrected P < 0.05).

The forward modeling technique allowed us to investigate the contribution of individual visual field maps and clusters in explaining the measured MEG signal, and at which latency during the visual response they did so.

## Discussion

We showed that our new forward modeling technique captures when different regions of the visual hierarchy are active, and how much they contribute to the visual response (Figs 2 and 3). This was achieved by harnessing the spatial accuracy of fMRI, building computational models identifying visual processing regions of interest, and utilizing MEG to determine response timing, with millisecond resolution (Figs 1 and 3). Our hierarchical processing results are consistent with invasive electrophysiology in both non-human primates and humans [2,25,26] and recent fMRI modeling [27]. However, compared to these previous approaches, our method uniquely provides non-invasive measures in humans that have high spatio-temporal resolution measures as well as full brain coverage.

While multimodal neuroimaging using fMRI and MEG has been previously established (e.g., [28–30]) our approach builds on forward models of neuronal population responses [15,16] to precisely capture when specific visual field maps contribute to visual responses. We leverage pRF modeling [17], which describes a core property of brain function [17,31,32] and is built upon well-established neuronal properties ([33]; for reviews see [34,35]). pRF fMRI estimates correlate strongly with estimates from invasive neurophysiology in non-human primates [36], invasive human electrodes [37,38] and visual perception [39], and have proven stable, robust and reproducible [40–42], supporting their use as a computational framework for linking fMRI and MEG.

### Model generalizability and robustness

We validated our model by demonstrating generalizability across (i) sensor types, (ii) stimulus types and (iii) number of predictors (Fig 1B-1D).

Our approach generalized across MEG sensors, i.e., magnetometers and planar gradiometers (Fig 1B), which differ in what they measure. Magnetometers detect the magnetic field at a point and are sensitive to both brain signals and distant noise. The planar gradiometers, on the other hand, measure the magnetic field gradient between two nearby points in two orthogonal directions on the sensor plane. This makes them more sensitive to local brain activity directly beneath the sensor and less affected by distant interference. Importantly, the underlying neural sources of the recorded fields/field gradients are the same for the two sensor types. The ability to generalize across these different types of sensors, therefore, highlights the capacity of the pRF model to capture sensor-independent neural activity.

Our approach generalized across stimuli, i.e., contrast-defined bar and circle stimuli (Fig 1C), although generalizability was somewhat lower across stimuli than sensor types. This is expected, as neural processing, including pRFs and single-neuron responses, varies with stimulus properties and task demands [43,44]. The larger bar stimuli, in particular, likely evoked more nonlinear responses such as compression, surround suppression, and oversaturation [45–48], which are not accounted for by our Gaussian model [17]. While advanced pRF models could improve generalizability [27,45,46,48], these findings also highlight the importance of aligning stimulus properties between fMRI and MEG. Nonetheless, our pRF model explained up to 66% of the response to a held-out stimulus, indicating it already provides a robust model of neural responses.

The estimated V1 time-course was robust to the number of other visual field maps and clusters included in the analyses (Fig 1D). This indicates generalizability along the number of regressors included and suggests that the regressors are orthogonal. Thus, the estimate of the time-course of a given region, does not depend on how many regions are estimated.

### Latency differences across visual field maps and clusters

The different response latencies across the visual hierarchy were consistent with invasive electrophysiology in both non-human primates and humans [2,25,26] and spatiotemporal fMRI modeling [27]. Compared to these previous approaches, our method uniquely combines non-invasive, high temporal resolution, with full brain coverage in humans. This finding highlights the potential of our approach as a non-invasive tool for capturing high-temporal resolution brain dynamics, applicable for a wide range of human experiments.

The cumulative approach allowed us to investigate the timing (or latencies) of the visual field maps and clusters without making prior assumptions and offers an overview of the entire time window during which the individual areas are active and contribute to visual processing. We do not interpret the hierarchical delay as “one area follows the other”. There is an intricate interplay between regions along the hierarchy, with a general hierarchical trend of increasing latencies [2,49] (see also [50]). Latencies may depend on the type of stimuli used [25,43,44,47], and other cognitive influences such as attention and task-demands [9,11]. Moreover, at each activation window we observed multiple responses indicative of an intricate interplay between regions [4,5]. Importantly, our approach enables future research to study how different tasks or cognitive states affect the contribution of different visual areas, and to do so with millisecond temporal resolution to detect any dynamic changes.

We found that V1 explained the most variance in most participants (Figs 2B and S3A-S3C). Based on the anatomy alone we would not expect V1 to dominate the signal: gain matrix values did not indicate higher sensitivity to V1 than to the other areas (S5 Fig), and V1 did not contain more cortical surface points than the other visual areas (S6 Fig). Instead, we suggest that V1’s observed dominance in our data could be due to the fact that we used low-level contrast defined visual stimuli that strongly activate early visual regions and its highly organized parallel processing architecture [51–54]. It could be that more complex stimuli would activate higher order areas more strongly [55], resulting in a different distribution of visual areas contributions than observed here.

### Limitations

Our approach for measuring pRFs and the manual identification of visual field maps and clusters is time consuming for both the participant and the researcher. While existing atlases may be used to reduce this burden [54,56,57], future experimental designs may also benefit from optimizing the number of stimulus repetitions. In the current study, the robustness and generalizability of our results across sensor types, stimulus types and number of regressors, suggests that a comparable or smaller number of repetitions may be sufficient for experiments with similar stimulus and task properties. In particular, designs that do not require data splitting and cross-validation may allow for fewer repetitions, reducing participant time in the scanner. More generally, statistical sensitivity in this framework depends not only on participant number, but also on the number of trials per participant, the signal-to-noise ratio, the stimulus design, and the analysis choices. In the present dataset, these factors were sufficient to detect V1 versus extrastriate latency differences, but not more subtle differences among extrastriate areas. Future studies targeting such finer-grained effects will likely require more data, either through additional participants, more trials, or both. Ultimately, the number of repetitions required will depend on the signal-to-noise ratio of the neural responses, which is shaped by stimulus characteristics and task demands of the experiment [58,59]. In its current implementation, the model is tailored to briefly presented static apertures; extending it to dynamic stimuli, such as a moving bar, would require generating time-resolved predictions that track the stimulus over time.

fMRI and MEG measure different neural signals: fMRI detects changes in blood oxygenation related to neural metabolism, while MEG records the magnetic fields generated by synchronized neural electrical activity. These differences ultimately limit the fusion of fMRI and MEG data. For example, fMRI may fail to detect neural activity beneath large draining veins due to vascular artifacts, whereas MEG can still capture the underlying signals [60]. Conversely, MEG may miss desynchronized activity because such patterns produce little net magnetic field, while fMRI can detect the associated metabolic changes [61]. Yet despite these differences, MRI-based pRFs explain a large portion of the MEG signals [15,16].

Improved head models and signal space separation to correct for head movements could further improve the quality of the results [22,62,63]. Although signal space separation preprocessing reduces the statistical independence between sensor types [64], it is still valuable to assess whether our model generalizes across different sensor representations of the same underlying neural activity. The fact that performance was preserved across sensor types suggests that the model captures features of the underlying signal rather than sensor-specific characteristics.

Additionally, our approach is adaptable to other modalities. For instance, optically pumped magnetometers (OPMs), offer advantages over traditional MEG systems by providing better signal-to-noise ratios and accommodating head movements [65,66]. Our approach can extend to EEG as well, which is more widely accessible than cryogenic MEG / OPMs, by using appropriate individual head conductivity models [30,67–69].

## Conclusion

Our approach estimates how individual cortical regions contribute to visual responses in humans. By combining fMRI and MEG with biologically-inspired models, we achieve temporal resolutions comparable to invasive electrophysiology while retaining the spatial specificity of functionally defined visual field maps. Our forward modeling relies on biologically inspired pRF models [17], which provide mechanistic insights into brain function [34,35]. Beyond bridging fMRI and MEG, the proposed approach enables us to pinpoint when specific cortical regions contribute to visual processing in individual humans, and how that timing changes with stimulus properties, task demands, or disease. Building on the successful extension of pRF models from vision to sensory [70,71], cognitive [9,72,73], and clinical domains, including ophthalmological, neurological, and psychiatric conditions [74–76], this framework opens new opportunities to study the timing of neural computations across these different domains and conditions. More broadly, it can be adapted to any biologically inspired computational model.

## Methods

### Ethics statement

The study was approved by the Scientific and Ethical Review Board (VCWE) of the Faculty of Behavior & Movement Sciences, Vrije Universiteit Amsterdam, and it was conducted in accordance with the ethical guidelines outlined in the Declaration of Helsinki (World Medical Association, 2000). All participants gave their written informed consent prior to the study.

### Experimental procedure and preprocessing

The full experimental procedure and preprocessing (Participants, Stimuli, Data acquisition and Data Preprocessing) is described in Eickhoff et al. [15]. In brief, five participants were measured in a separate fMRI and MEG session. All participants were screened for fMRI and MEG compatibility, had normal or corrected-to-normal visual acuity, and did not have a history of neurological or psychiatric disorders. During both sessions, contrast-defined stimuli were presented, eliciting responses from similar neuronal populations across sessions, and the participants were fixating on the center of the screen the entire time. Both fMRI and MEG data were minimally preprocessed before being fed into our analysis pipeline.

**Fig 4:**
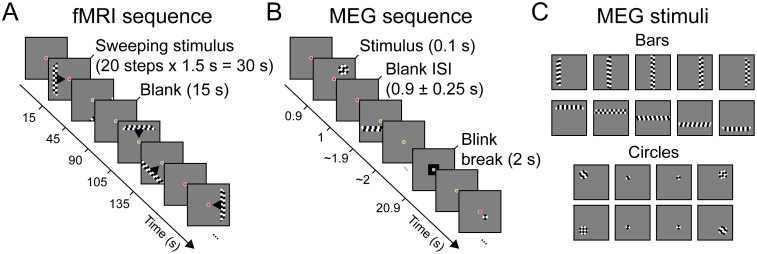
Stimulus sequence. **A**. A contrast-defined bar stimulus, interleaved by mean-luminance blanks, mapped the visual space in eight different directions while measuring fMRI. The stimulus sequence is a standard procedure to estimate pRFs (see [17]). **B**. Bar and circle shapes (shown in C) containing the same contrast-defined checkerboard were briefly (100 ms) shown in semi-random order, while measuring MEG. The stimulus trials were interleaved by mean-luminance blanks and blink periods. **C**. The eighteen MEG stimulus shapes, classified in two groups: ten *bars* (five vertical bars and five horizontal bars), and eight *circles*. The bars were either 0, 1.82 or 3.06 deg from screen center, the circles either 1.82 or 3.06 deg from center in the four visual quadrants, with a diameter of 1.25 and 3.06 deg respectively.

The functional MRI data was interpolated from voxel to vertex space to estimate pRFs on the cortical surface. The fMRI checkerboard stimuli (Fig 4A) and vertex data was used to estimate pRFs as described in Dumoulin & Wandell [17], and we subsequently predicted the pRFs responses to the MEG stimuli that were presented during the MEG session (Fig 4B and 4C).

The MEG stimuli were identical to those used by Eickhoff et al. [15] and comprised 18 contrast-defined apertures (Fig 4C): five vertical bars, five horizontal bars, and eight circles (overall stimulus extent 4.9°). Bars were 1.25° wide and presented at 0°, 1.82°, or 3.06° eccentricity (five locations per orientation), whereas circles were presented in one of eight quadrant-specific locations (four quadrants × two eccentricities: 1.82° and 3.06°) with diameters of 1.25° and 2.5° at the small and large eccentricity, respectively. Stimuli were presented for 100 ms and followed by a mean-luminance screen (900 ms ± 250 ms) to evoke event-related fields (ERFs); during the 100 ms presentation, the aperture remained static while the checkerboard rows moved in opposite directions. Participants performed a fixation color-change task throughout.

The MEG preprocessing followed the pipeline described in Eickhoff et al. [15], but we summarize the essential steps here. MEG data were acquired at 1000 Hz with an online high-pass filter at 0.10 Hz and low-pass filter at 330 Hz, using internal active shielding. Raw data were first preprocessed with temporal signal space separation (tSSS [77]; MaxFilter Software Elekta Neuromag, Oy, version 2.2.15) to reduce environmental noise and compensate for head movement; malfunctioning or noisy channels were interpolated during this step (mean 5.5 of 306 channels). Stimulus onsets were identified using a photodiode recorded alongside the MEG data, and periods containing missed screen flips were discarded. Data were then epoched from -100–600 ms relative to stimulus onset, yielding on average 186.2 epochs per stimulus, and each epoch was baseline corrected using the -100–0 ms prestimulus interval. No further preprocessing or filtering steps were applied before computing event-related fields (ERFs) used in the modeling analyses, however, subsequent analyses were restricted to the 0–400 ms post-stimulus window.

The MEG data and the anatomical MRI scan were used to create the participants’ gain matrix. The gain matrix (MEG sensors × fMRI vertices) contains the weights of the vertices’ contribution to the signal measured with the MEG sensors. We estimated the gain matrix with the overlapping spheres (OS; [22]) model, and constrained the dipoles to be perpendicular to the cortical surface.

The fMRI-based pRFs and gain matrix were used, together with the MEG data, to perform our forward modeling approach.

### Forward modeling approach

**Fig 5:**
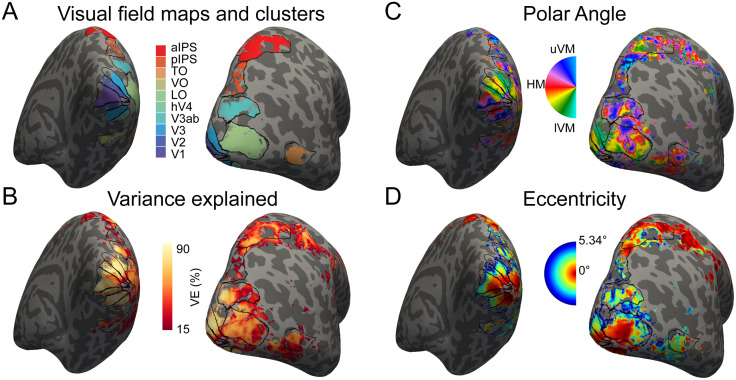
Visual field maps and clusters. The left column in each panel shows a medial posterior view of participant 1’s cortical surface. The right column is a lateral posterior view. Only vertices included in the analysis are color-coded. **A**. Visual field maps and clusters that were defined by hand, based on the visual field maps and clusters shown in row two to four. **B**. How much variance a given pRF explained in the fMRI data. **C**. The polar angle in visual space associated with a given pRF, ranging from the upper vertical meridian (uVM), through the horizontal meridian (HM) to the lower vertical meridian (lVM). **D**. The eccentricities in visual space of the pRFs; 5.34 deg was the maximum eccentricity at which the fMRI stimuli were presented.

To estimate spatiotemporal responses to visual stimuli in the visual system with millisecond resolution, we applied a forward model that converts visual field maps-specific predictions to MEG sensor level, allowing us to estimate how much a given visual field maps contributes to the measured MEG response. In brief, fMRI was used to estimate participants’ pRFs and define visual field maps (Figs 5 and 1A Step 1) and MEG sensor responses were collected in response to MEG stimuli (Figs 4C and 1A Step 2). Next, we predicted how the pRFs would respond to the same MEG stimuli (Figs 4C and 1A Step 3). Then, we confined the predictions to one of the ten defined visual field maps or clusters (VFMs). For that, the predictions were converted to sensor-level by applying VFM-masked gain matrices (Fig 1A Step 4), and we computed how much each of the predicted responses explained the measured MEG data in a cross-validated manner (Fig 1A Step 5). To demonstrate the generalizability and reliability of the approach, we performed three method validation tests, namely 1) testing whether the model generalized over MEG sensor types (magnetometers vs planar gradiometers), and 2) stimulus shapes (bars vs circles), and 3) testing whether the model results were robust to the number of predictors used in the model. The analysis steps are explained in more detail below.

#### Step 1: Estimate pRFs and visual field maps and clusters.

To estimate the participants pRFs, we measured fMRI while participants viewed contrast-defined bar stimuli that moved across the visual field (Fig 4A). For each cortical location (called ‘vertex’), a pRF was estimated as previously described in Dumoulin & Wandell [17]. A brief overview follows:

The accumulated receptive fields of a population of neighboring neurons can be approached as a 2D Gaussian in visual space. A range of these 2D Gaussian models with different visual locations were constructed, and their responses to the bar stimuli predicted. The predicted time-courses are the result of matrix multiplication of the 2D binarized stimulus and the 2D Gaussian. The predicted time-courses are then fitted to the measured time-courses to the same stimuli for a given cortical location, and the Gaussian that fits best was selected as the estimated population receptive field (pRF) for that vertex. The pRF thus described the part of the visual field to which a cortical location responds the most. For the following analysis steps, we only used vertices with pRFs that had fits with variance explained (VE) of at least 15%. We also excluded vertices that were located under a vein, and where the pRF eccentricity was located outside the visual space in which the stimuli were presented.

We then defined visual field maps and clusters for each participant by hand (Fig 5A), based on the anatomical landmarks, and pRF polar angle and eccentricity estimates (Fig 5B-5D). Visual field maps are defined as contiguous regions of cortex in which there exists a precise, systematic, and non-redundant mapping between locations in the visual field and specific locations on the cortical surface. In these regions, neighboring points in the visual field are represented by neighboring points on the cortex, preserving the topography and layout of visual space [21]. We identified four visual field maps: V1, V2, V3, hV4, and six visual field map clusters: V3ab, LO, VO, TO, posterior IPS (pIPS) and anterior IPS (aIPS). Visual field map *clusters* are spatially contiguous groups of visual field maps in the cortex that share common organizational features, such as a shared foveal representation. Note that we could have merged certain regions together (for example, posterior and anterior IPS), or separate them even further. However, for the purpose of this method, the current level of specificity matched other pRF modeling papers [25,45].

#### Step 2: Measure MEG responses to MEG stimuli.

We measured MEG data with 306 sensors at 102 sensor locations, while participants viewed different bar and circle stimuli (Fig 4C). At each location, the MEG system records three channels: one magnetometer measuring the absolute component of the magnetic field and two orthogonally oriented planar gradiometers measuring spatial gradients along two perpendicular tangential directions (Fig 1B).

#### Step 3: Predict pRFs’ responses to MEG stimuli.

We next predicted how the pRFs on the participants’ cortical surface respond to each of the MEG stimuli shown. The predicted response is the spatial overlap between the 2D binarized stimulus and the 2D pRF models, calculated as matrix multiplication. This step resulted in a matrix of (vertices × stimuli), containing each pRF’s response to each of the eighteen stimuli (Fig 1A Step 3).

#### Step 4: Convert visual field maps- and clusters’ pRF responses to MEG sensor-level.

To identify whether the pRFs estimated on the cortical surface can explain the MEG data, the pRFs responses need to be converted to the MEG measurement space. That is, while the pRFs predictions live in cortical space, the MEG data was measured at the sensor-level. The gain matrix determines how much each vertex contributes to a given sensor, hence multiplying the pRF predictions with the gain matrix converts the predicted cortical values to sensor-level: matrix-multiplying the gain matrix (sensors × vertices) with the cortical pRF predictions (vertices × stimuli), scales each vertex’ response by the appropriate amount for each MEG sensor, resulting in a matrix of (sensors × stimuli).

We do not expect that each visual field map or cluster (VFM) contributes the same amount at a given timepoint during the visual response. Instead, we expect that distinct visual regions would reveal different response time-courses. Early visual regions should respond early and receive feedback later in time. While higher order areas respond later as the signal needs time to travel up the cortical hierarchy first. We thus created VFM-specific predictions by letting only the vertices belonging to a given area weigh into the predicted sensor-level responses. More specifically, we created ten VFM-masked gain matrices (each sensors × vertices), in which the weights of all vertices outside a specific area were set to zero, and then multiplied the VFM masks with the gain matrix for the participant. Thus, only the given visual areas’ vertices now contributed to the sensor-level prediction. By matrix-multiplying each masked gain matrix with the vertex’ responses, we thus obtained ten VFM-specific sensor predictions (Fig 1A Step 4), each with shape (sensors × stimuli).

#### Step 5: Compare measured and predicted responses.

Lastly, we calculated how well each VFM-specific prediction fitted the MEG data using cross-validated ridge regression, for each of the timepoints of the visual response after stimulus onset. We opted for ridge regression since it allows for penalization of beta weights of correlated VFM-predictors.

For the cross-validation procedure, we obtained four sets of average responses (referred to as ‘event related field’ (ERF) responses) from the MEG data (Fig 1A Step 2 bottom). The averaging gets rid of unrelated noise while preserving stimulus-related responses in the signal. The averages were drawn from four random splits of trials per stimuli, resulting in four sets of (sensors × stimuli × timepoints). One of the sets was left out, and was used later as independent evaluation of how well the model predictions fit never-seen data (test set). The three other sets were used in a three-fold cross-validation method to find the parameters that resulted in the best fit between the ten VFM-predictors and the measured data.

Before the data matrices were fed into the ridge regression, the datapoints across all sensors and stimuli were concatenated to a big matrix of (sensors*stimuli) for both predictions and measured data. The VFM-predictions were thus a matrix of (sensors*stimuli) × VFMs, and the measured data for a specific timepoint *t* was (sensors*stimuli).

To perform ridge regression at a given timepoint, we implemented the *fracridge* toolbox in a cross-validated manner [24]. This procedure ensures the appropriate level of regularization is applied by reparameterizing the regression based on a given ratio (gamma) between the regularized and unregularized L2-norm coefficients. For a range of ratios (gammas) from 0.1 to 1 in steps of 0.1 the toolbox tested which ratio and accompanying regularization parameter (alpha) resulted in the best fit in a three-fold cross-validated manner: For a given gamma, one of the three datasets was left out for testing. The other two datasets were used to obtain the alpha parameter and beta values for each predictor. The betas were fixed and applied to the predictions to test on the left-out third set, obtaining the goodness of fit for that particular fold. This was repeated for the other two datasets. The three parameter sets resulting from this were then averaged, and the whole procedure repeated for the other gamma ratios. The gamma value and belonging parameters resulting in the highest goodness of fit were chosen, and applied to the predictions of the fourth, never-seen, test set, and the goodness of fit was computed. This goodness of fit value represents how well the full model (all predictors) explained the measured data at a given timepoint. We repeated this for all timepoints (0–400 ms) (Fig 1A Step 5). In the present dataset, regularization was low when visual signal was present (S7 Fig).

Finally, since we were interested in the individual contribution of the predictors (i.e., the VFMs) in explaining the measured data, we calculated how much variance (VE) each VFM explained in the data as follows:


$$\begin{array}{c} {VE}_{VFM}=\text{1}\text{0}\text{0}*\;\left(\text{1}-\;\left(\frac{\sum {\left(y_{test}-\;{\hat{y}}_{all}\right)}^{\text{2}}}{\sum {\left(y_{test}-\;{\hat{y}}_{other}\right)}^{\text{2}}}\right)\right) \end{array}$$ (1)

where $y_{test}$ contains ERF values (sensors*stimuli) at timepoint t of the test set, ${\hat{y}}_{all}=\;\sum_{vfm}\left({\hat{y}}_{vfm}*\;{\beta \text{1}}_{vfm}\right)$, and ${\hat{y}}_{other}=\;{\hat{y}}_{all}-\left({\hat{y}}_{VFM}*\;{\beta \text{1}}_{VFM}\right)$.

To quantify the latencies of the individual visual field maps- and clusters’ responses, we used a cumulative approach that takes the total history of the variance explained time-course into account and thus characterizes the entire ‘activation window’ of an area. This approach avoids assumptions and experimenters choices on parameters that would be necessary for other approaches such as peak detection.

For the cumulative approach, we calculated the normalized cumulative VE curves based on each variance explained time-course (S3B Fig). For that, we set timepoints with negative variance explained to zero and calculated each timepoint’s cumulative VE value as the sum over its current value and all past values up to that latency and the cumulative time-course was normalized to values between 0 and 1. We then examined the timepoints at which the normalized cumulative VE reached 25%, 50% and 75%.

### Method validation

We performed three validation tests. We examined, 1) how well the model generalized across the different sensor types of the MEG scanner (magnetometer vs planar gradiometers), 2) how well it generalized across the stimulus types used (bars vs circles), and 3) how robust the results were to the number of predictors (VFMs) included in the model.

#### Sensor type validation.

The MEG system used to measure the data holds two sensor types, which have different signal sensitivity profiles [78]. We wanted to test whether the model generalizes across these two sensor types. Instead of using all sensors for cross-validated fitting for a given train-test fold, we only used one sensor type for model training. The model performance was then evaluated on the left-out sensor type. We left out each sensor type once, resulting in two variance explained time-courses. Within each train-test combination, we used the same cross-validated three-fold procedure, i.e., the concatenated data of the ‘train’ sensor types was split into three sets to find the optimal regularization. Below, we report the final goodness of fit of the full model, calculated as:


$$\begin{array}{c} {VE}_{full}=\text{1}\text{0}\text{0}*\left(\text{1}-\;\left(\frac{\sum {\left(y_{test}-\;{\hat{y}}_{all}\right)}^{\text{2}}}{\sum {\left(y_{test}\right)}^{\text{2}}}\right)\right) \end{array}$$ (2)

where $y_{test}$ contains ERF values (sensors*stimuli) at timepoint t of the test set and ${\hat{y}}_{all}=\;\sum_{vfm}\left({\hat{y}}_{vfm}*\;{\beta \text{1}}_{vfm}\right)$. We report the Pearson correlation coefficient between the train-test results to quantify the stability across sensor type fits.

#### Stimulus type validation.

We tested the model’s generalizability across the responses to the different stimulus-shapes used. The two stimulus types were the ten bars and the eight circular stimuli (Fig 4C). Again, we used only one of the stimulus shapes for training of the model, and used the left-out stimulus type to evaluate the fit. We used the same cross-validated three-fold ridge regression procedure for each combination of train-test stimulus types, and again reported the final goodness of fit of the full model (Equation 2) for each fold and the correlation coefficient across these train-test folds.

#### Robustness to number of visual field maps and clusters.

The robustness of visual-fits to the number of predictors (i.e., the visual field map and clusters; VFMs) in the model was examined by evaluating models with different numbers of predictors. We focused on the V1 time-course and its stability as it resulted in the best variance explained across most participants (Fig 2). For this, we used all stimuli and sensors’ data; however, the predictor matrices now originated from different numbers of VFMs. That is, first we used only the predicted values from V1, then incrementally added each VFM until the model contained the full ten VFMs. We performed the same three-fold cross-validation procedure as described above, leaving out a fourth, independent set of data to test the model. We reported the V1 prediction fit (as calculated in Equation 1), for each of the models tested and to quantify the stability, and reported the correlation coefficient between the V1-only fit to the other nine model fits. The effect of removing V1 (the main predictor; Fig 2) on the stability of the fits of the other VFMs was examined by excluding V1 from the model fits. The results can be found in S2 Fig.

### Statistical analysis

For the statistical analysis we performed the cross-validated fitting procedure 120 times using 120 random averages of the MEG data, referred to as cross-validation folds. We quantified activation onset for each visual field map and cluster (VFM) as the time point at which a fold’s normalized cumulative cross-validated variance explained reached 25% of its total within the 0–400 ms post-stimulus window. For each participant and VFM, this procedure was applied to all 120 cross-validation folds, yielding a distribution of activation onset estimates per area.

We performed statistical comparisons of activation onset latencies within participants, comparing each extrastriate VFM against V1. Differences were assessed using a paired Fisher permutation test based on 10,000 permutations, applied to the paired onset distributions across folds. Resulting p-values were Bonferroni-corrected for multiple comparisons across visual field maps and clusters. Extrastriate areas with a corrected p-value < 0.05 were considered to show a significant latency difference relative to V1 and are indicated by opaque shading in the figure; non-significant comparisons are shown with transparent shading and marked ‘n.s.’.

## Supporting information

S1 Fig Method validation results for all participants. A. Cross-validated variance explained (*Methods* Equation 2) for the model trained on one sensor type and tested on the left-out sensor type’s data for all participants (rows). The Pearson correlation coefficient of the two time-courses (shown in upper right corner) was high, ranging from  .93 to  .98 across participants, indicating our model generalized across sensor types. B. Cross-validated variance explained for the model trained on one stimulus type and tested on the left-out stimulus type’s data (bars vs circles) for all participants (rows). C. V1’s cross-validated variance explained time-course (*Methods* Equation 1) for models with different numbers of visual field maps and clusters for all participants (rows); ranging from including all ten visual field maps and clusters (black line), to only including V1 (yellow line). Presented r range (upper right corner) reflects the relationship of the V1-only model fit to the other nine model fits. The high r (ranging from 0.83 to 1.00) indicates that V1’s fit was stable across different numbers of visual field maps and clusters included in the model.
(TIF)

S2 Fig Visual field maps and clusters’ variance explained fitted with and without V1 regressor. A. For each participant (P1 to P5) and visual field map and cluster (rows): cross-validated variance explained time-course resulting from two model fits: when V1 was included in the model (black lines; same model fit as in main analysis), and when V1 was excluded from the model fit (colored lines). In each panel the correlation between the two fits (“Fit correlations”) are indicated in black. All Fit correlations had a p-value of <.001 (N = 398), except for V2 in P2 (*p = .28. For most visual field maps and clusters across participants these correlations were high, indicating that the fits were stable and independent of V1’s inclusion in the model. Some fits changed, which is reflected in lower correlation values; these are highlighted in bold (r ≤ .85 or ≥ -.85). The low fit correlations often occurred together with high “Prediction correlations” (indicated in purple, and highlighted in bold if r ≥ .25 or ≤ -.25); which are the correlations between the respective visual field’s predicted values and V1’s predicted values, indicating that the regressors cannot be distinguished well by the model. All prediction correlations had a p-value of <  .001 (N = 398), except those with stars; deviating p-values in order of participants, top to bottom are: P2 V3: *p* = .349, P2 V3ab: *p* = .531, P2 pIPS: *p* = .002, P2 aIPS: *p* = .513; P3 V3ab: *p* = .005, P3 VO: *p* = .288; P5 V3ab: *p* = .019, P5 hV4: *p* = .047, P5 LO: *p* = .013, P5 TO: *p* = .001, P5 aIPS: *p* = .019. B. Summary plot of the Fit and Prediction correlations plots. Each color corresponds to the visual field map or cluster; participants 1–5 had markers: star, circle, square, downward triangle, upward triangle, respectively. Most Fit correlations were high (above 0.85), indicating stable fits independent of V1 in- or exclusion. Lower Fit correlations were related to higher Prediction correlations.
(TIF)

S3 Fig Variance explained time-courses of all participants. A. Cross-validated variance explained time-courses of each visual field map and cluster (see middle inset for color code) for all participants (rows P1-P5) from 0 to 400 ms after stimulus onset. The gray shaded area indicates the period when the stimulus was shown. B. Normalized cumulative variance explained (VE) for each visual field and participant.
(TIF)

S4 Fig Using peak detection for latency quantification. A. For each participant (P1 to P5) and visual field map and cluster (rows), we show the peaks found for two peak detection methods (markers ‘v’ and ‘^’ for method 1 and 2, respectively), on top of the cross-validated variance explained (VE) time-courses for each visual field map and cluster (note the different vertical scales). For both methods we smoothed the VE time-courses with a 20 ms moving window, and identified local maxima with a width of at least 10 ms, but we applied different thresholds: for method 1 we considered all peaks with variance explained above 0; for method 2 we only considered peaks with a variance explained of at least 50% of the maximum variance explained. For comparison, below each VE time-course are the latency window results found in our main analysis; vertical ticks mark the latencies at which normalized cumulative variance explained reached 25, 50 and 75% of the total across the shown 400-ms time-window. B. Peaks and cumulative time windows for all subjects (P1 to P5 from top to bottom) for each visual field map or cluster (rows). For method 1, the average first peak detected was 81 and 112 ms for V1 and extrastriate regions, respectively; for method 2, the average peak was 81 for V1 and 141 ms for extrastriate regions. Both peak detection approaches resulted in earlier latencies than the activation time window determined by the cumulative approach with an average 25% percentile onset of 89 and 151 ms for V1 and extrastriate regions, respectively.
(TIF)

S5 Fig Gain values for visual field maps and clusters. Absolute gain values averaged over 102 magnetometers and 204 planar gradiometers, for each visual field map/cluster and participant separately.
(TIF)

S6 Fig Number of vertices per visual field map/cluster included in the analysis for each of the five participants. (TIF)

S7 Fig Full model ridge regression cross-validation outcomes. For all five participants (columns). *Top row*: Full model variance explained (VE) time-courses for all participants calculated as in Equation 2. This time-course shows how much all visual field maps and clusters together explain the measured MEG data. *Middle row*: Best average gamma (ratio) found for each timepoint with the three-fold cross-validation procedure. A value of 1 corresponds to no difference between the regularized and non-regularized outcomes. High gamma values correspond to low alpha (regularization parameter) values, as shown in the *bottom row*: High alpha values indicate high regularization (high punishment of beta values, for example if predictions are correlated). An alpha value of 0 corresponds to no regularization, so ordinary least squares. These results indicate that when the signal is present, generally no regularization was required.
(TIF)
