# Supplementary figures and images for: Non-invasive mapping of the temporal processing hierarchy in the human visual cortex
Source: PLoS Comput Biol. 2026 Jul 10;22(7):e1014434. doi: 10.1371/journal.pcbi.1014434 (PMC13379088; doi:10.1371/journal.pcbi.1014434)

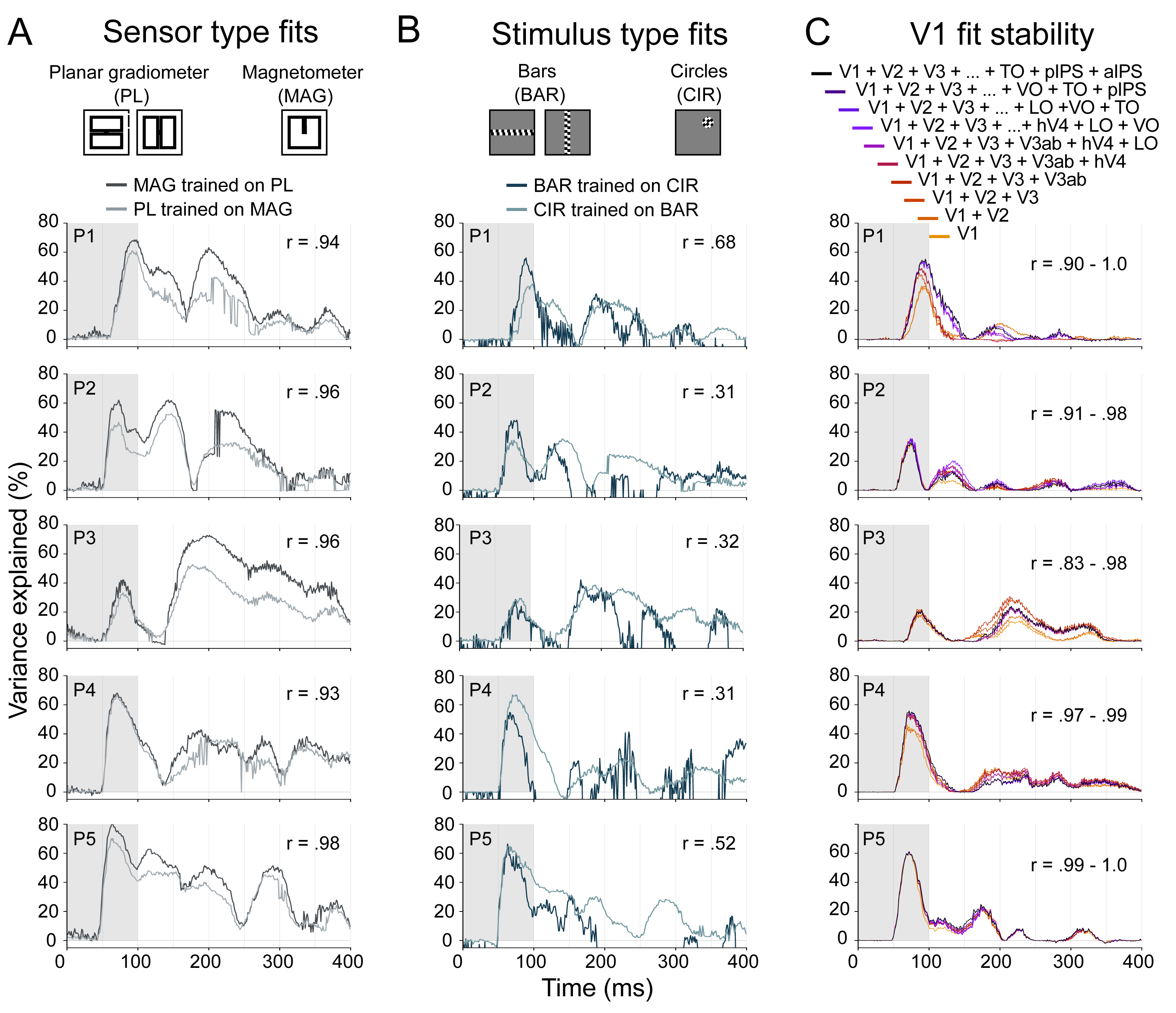

Supplement: S1 Fig — A. Cross-validated variance explained (Methods Equation 2) for the model trained on one sensor type and tested on the left-out sensor type’s data for all participants (rows). The Pearson correlation coefficient of the two time-courses (shown in upper right corner) was high, ranging from .93 to .98 across participants, indicating our model generalized across sensor types. B. Cross-validated variance explained for the model trained on one stimulus type and tested on the left-out stimulus type’s data (bars vs circles) for all participants (rows). C. V1’s cross-validated variance explained time-course (Methods Equation 1) for models with different numbers of visual field maps and clusters for all participants (rows); ranging from including all ten visual field maps and clusters (black line), to only including V1 (yellow line). Presented r range (upper right corner) reflects the relationship of the V1-only model fit to the other nine model fits. The high r (ranging from 0.83 to 1.00) indicates that V1’s fit was stable across different numbers of visual field maps and clusters included in the model. (TIF) [file pcbi.1014434.s001.tif]

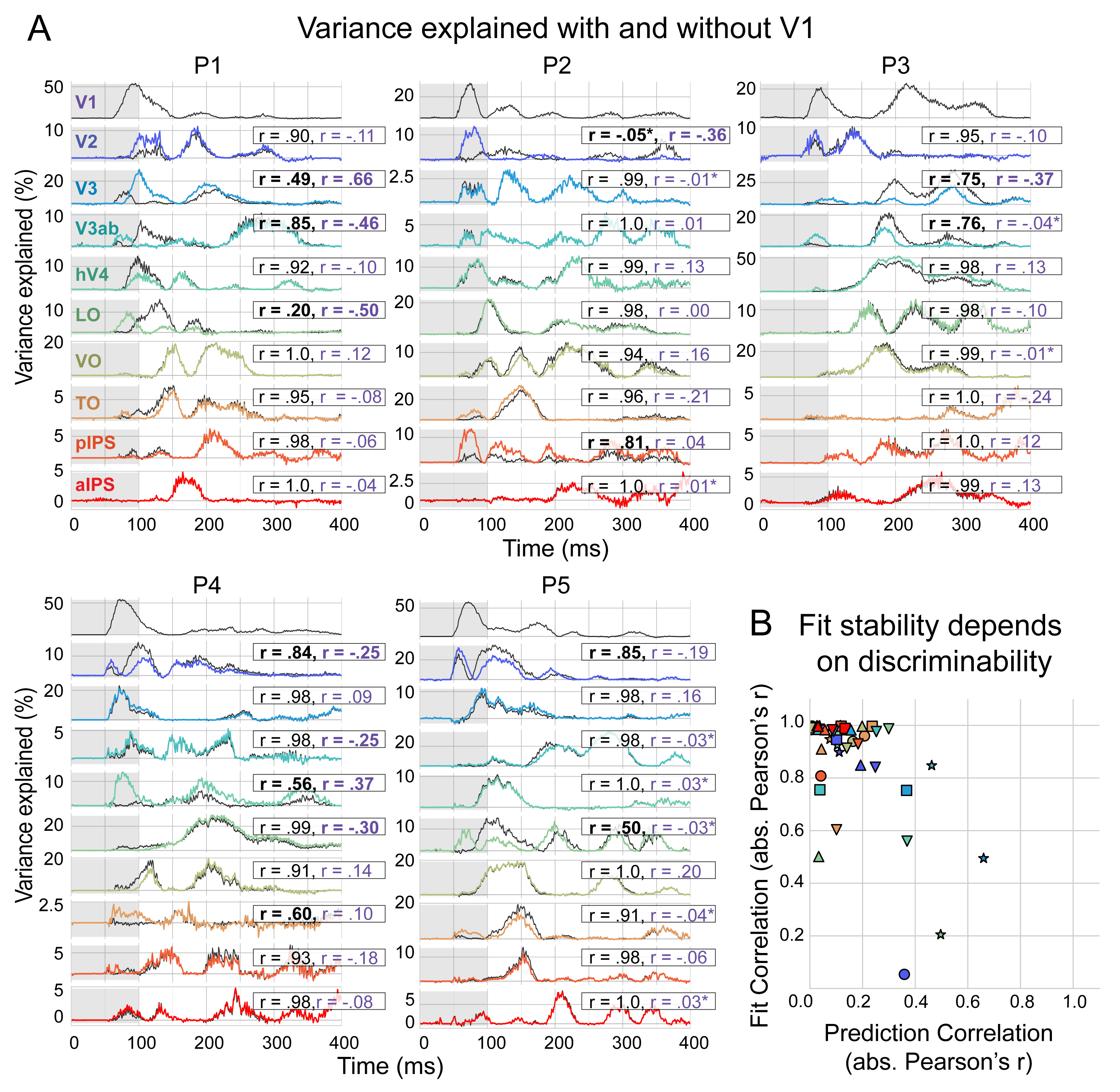

Supplement: S2 Fig — A. For each participant (P1 to P5) and visual field map and cluster (rows): cross-validated variance explained time-course resulting from two model fits: when V1 was included in the model (black lines; same model fit as in main analysis), and when V1 was excluded from the model fit (colored lines). In each panel the correlation between the two fits (“Fit correlations”) are indicated in black. All Fit correlations had a p-value of <.001 (N = 398), except for V2 in P2 (*p = .28. For most visual field maps and clusters across participants these correlations were high, indicating that the fits were stable and independent of V1’s inclusion in the model. Some fits changed, which is reflected in lower correlation values; these are highlighted in bold (r ≤ .85 or ≥ -.85). The low fit correlations often occurred together with high “Prediction correlations” (indicated in purple, and highlighted in bold if r ≥ .25 or ≤ -.25); which are the correlations between the respective visual field’s predicted values and V1’s predicted values, indicating that the regressors cannot be distinguished well by the model. All prediction correlations had a p-value of < .001 (N = 398), except those with stars; deviating p-values in order of participants, top to bottom are: P2 V3: p = .349, P2 V3ab: p = .531, P2 pIPS: p = .002, P2 aIPS: p = .513; P3 V3ab: p = .005, P3 VO: p = .288; P5 V3ab: p = .019, P5 hV4: p = .047, P5 LO: p = .013, P5 TO: p = .001, P5 aIPS: p = .019. B. Summary plot of the Fit and Prediction correlations plots. Each color corresponds to the visual field map or cluster; participants 1–5 had markers: star, circle, square, downward triangle, upward triangle, respectively. Most Fit correlations were high (above 0.85), indicating stable fits independent of V1 in- or exclusion. Lower Fit correlations were related to higher Prediction correlations. (TIF) [file pcbi.1014434.s002.tif]

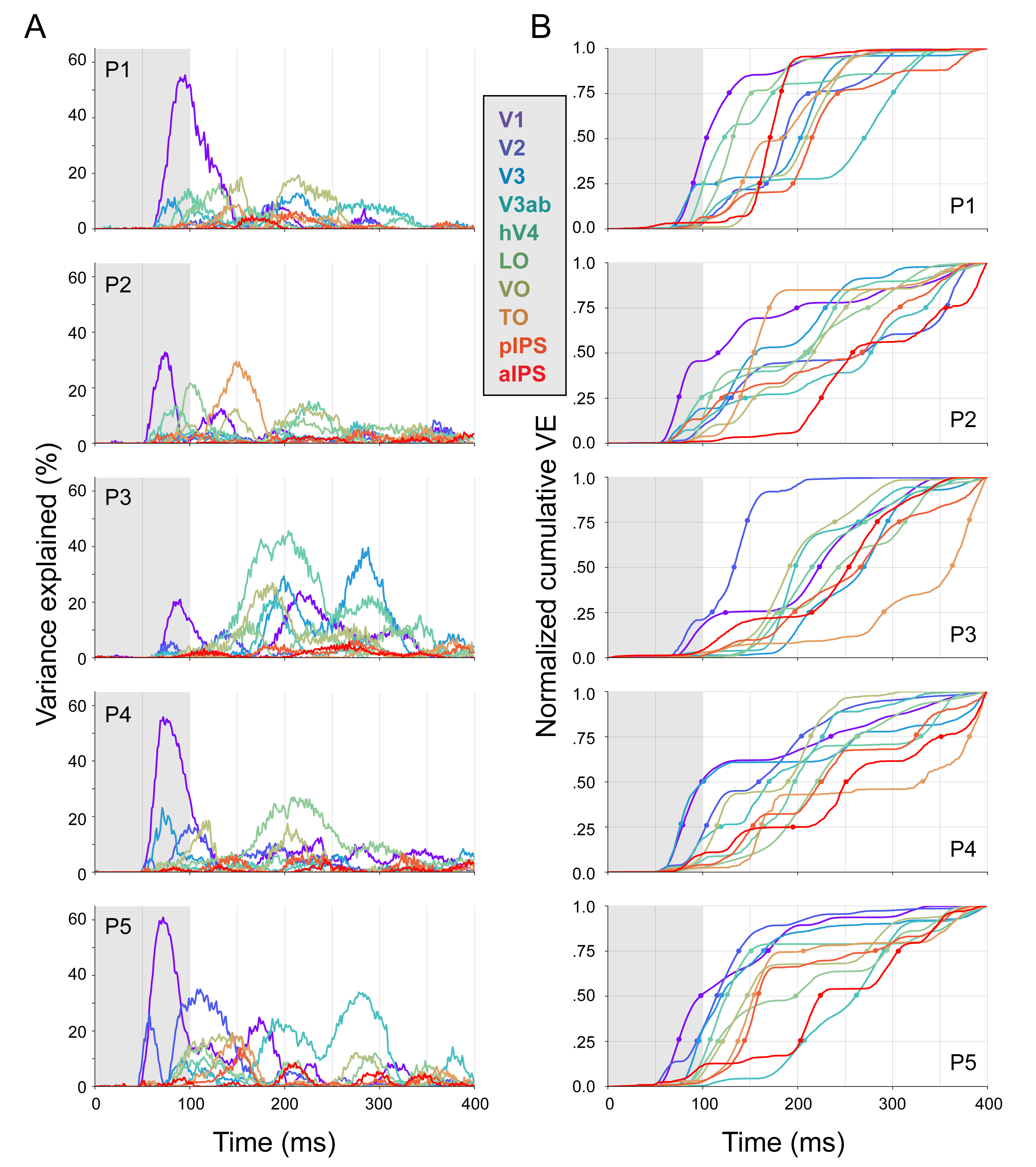

Supplement: S3 Fig — A. Cross-validated variance explained time-courses of each visual field map and cluster (see middle inset for color code) for all participants (rows P1-P5) from 0 to 400 ms after stimulus onset. The gray shaded area indicates the period when the stimulus was shown. B. Normalized cumulative variance explained (VE) for each visual field and participant. (TIF) [file pcbi.1014434.s003.tif]

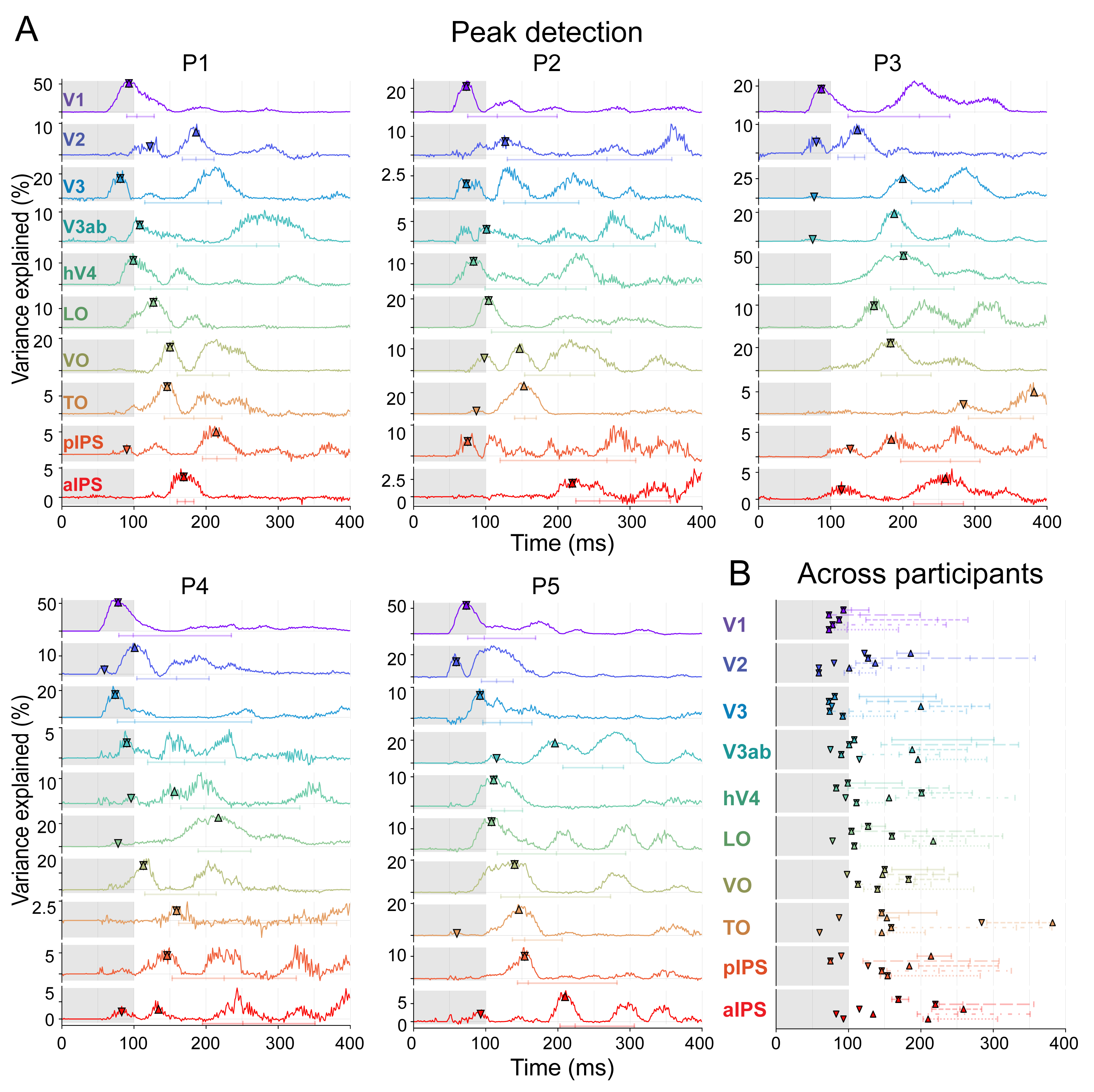

Supplement: S4 Fig — A. For each participant (P1 to P5) and visual field map and cluster (rows), we show the peaks found for two peak detection methods (markers ‘v’ and ‘^’ for method 1 and 2, respectively), on top of the cross-validated variance explained (VE) time-courses for each visual field map and cluster (note the different vertical scales). For both methods we smoothed the VE time-courses with a 20 ms moving window, and identified local maxima with a width of at least 10 ms, but we applied different thresholds: for method 1 we considered all peaks with variance explained above 0; for method 2 we only considered peaks with a variance explained of at least 50% of the maximum variance explained. For comparison, below each VE time-course are the latency window results found in our main analysis; vertical ticks mark the latencies at which normalized cumulative variance explained reached 25, 50 and 75% of the total across the shown 400-ms time-window. B. Peaks and cumulative time windows for all subjects (P1 to P5 from top to bottom) for each visual field map or cluster (rows). For method 1, the average first peak detected was 81 and 112 ms for V1 and extrastriate regions, respectively; for method 2, the average peak was 81 for V1 and 141 ms for extrastriate regions. Both peak detection approaches resulted in earlier latencies than the activation time window determined by the cumulative approach with an average 25% percentile onset of 89 and 151 ms for V1 and extrastriate regions, respectively. (TIF) [file pcbi.1014434.s004.tif]

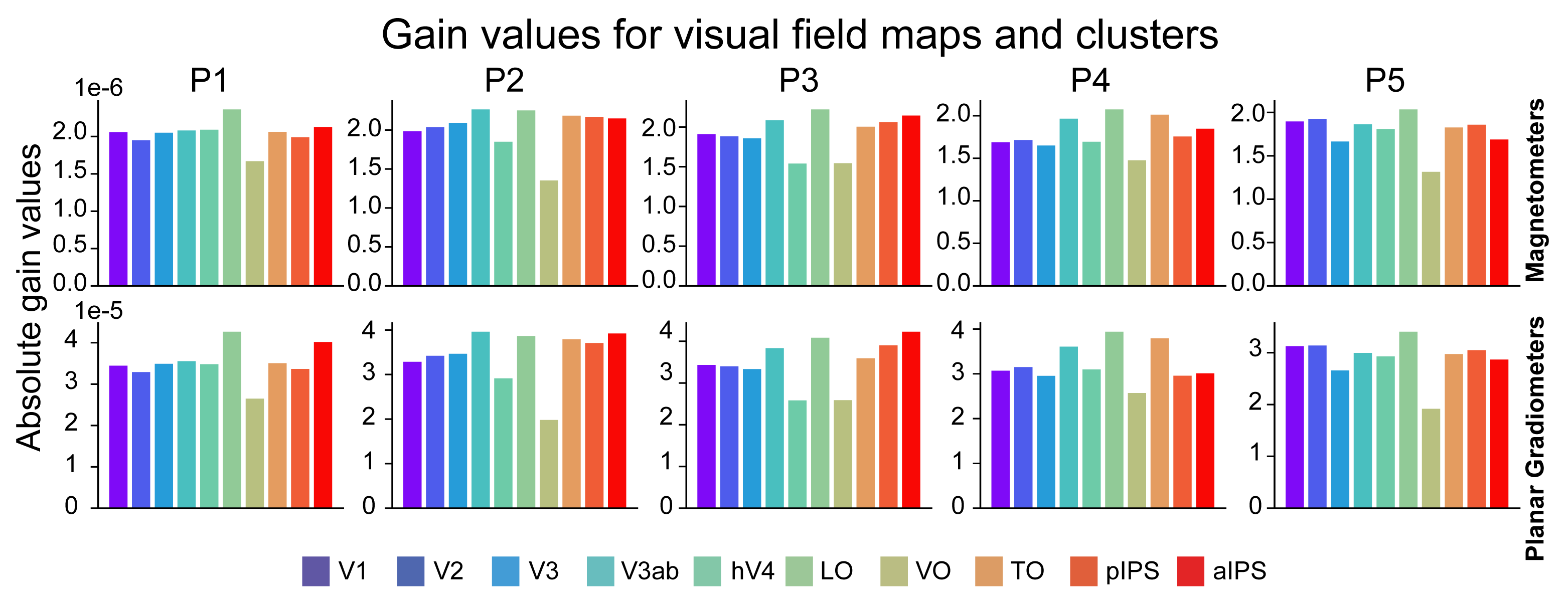

Supplement: S5 Fig — Absolute gain values averaged over 102 magnetometers and 204 planar gradiometers, for each visual field map/cluster and participant separately. (TIF) [file pcbi.1014434.s005.tif]

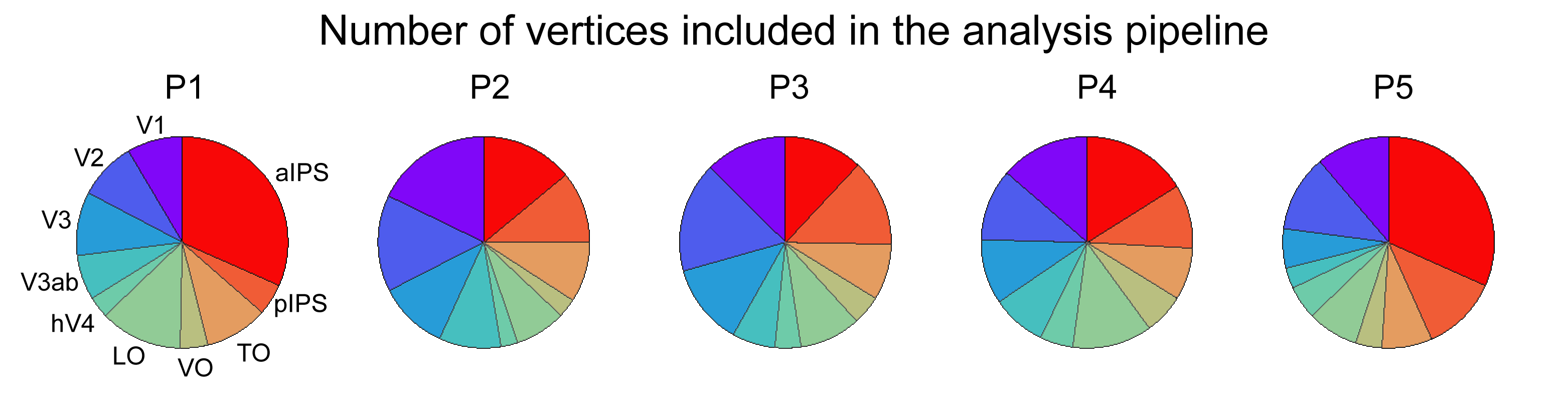

Supplement: S6 Fig — (TIF) [file pcbi.1014434.s006.tif]

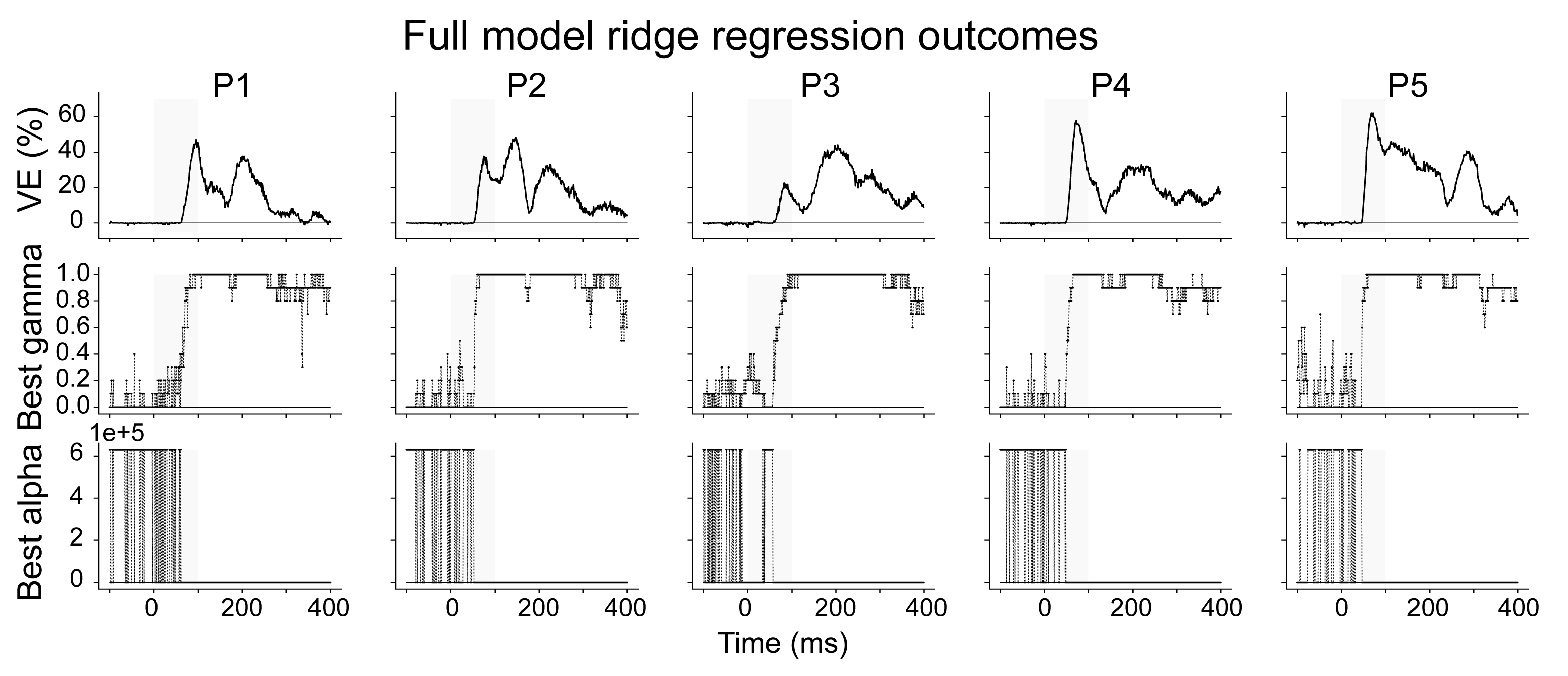

Supplement: S7 Fig — For all five participants (columns). Top row: Full model variance explained (VE) time-courses for all participants calculated as in Equation 2. This time-course shows how much all visual field maps and clusters together explain the measured MEG data. Middle row: Best average gamma (ratio) found for each timepoint with the three-fold cross-validation procedure. A value of 1 corresponds to no difference between the regularized and non-regularized outcomes. High gamma values correspond to low alpha (regularization parameter) values, as shown in the bottom row: High alpha values indicate high regularization (high punishment of beta values, for example if predictions are correlated). An alpha value of 0 corresponds to no regularization, so ordinary least squares. These results indicate that when the signal is present, generally no regularization was required. (TIF) [file pcbi.1014434.s007.tif]
