# Supplementary material for: Non-invasive mapping of the temporal processing hierarchy in the human visual cortex
Source: PLoS Comput Biol. 2026 Jul 10;22(7):e1014434. doi: 10.1371/journal.pcbi.1014434 (PMC13379088; doi:10.1371/journal.pcbi.1014434)
Supplement: Attachment — Submitted filename: Response to Reviewers.pdf [file pcbi.1014434.s009.pdf]

Response to reviewers

Dear Yuanning Li and Hugues Berry,

We thank you and the reviewers for the thoughtful and constructive feedback on our manuscript "Non-invasive mapping of the temporal processing hierarchy in the human visual cortex". We have carefully considered all comments and made revisions accordingly, including new statistical analyses and a new figure (Fig 3C). Below we provide a point-by-point response, with reviewer comments reproduced in green italics, followed by our responses. We believe these changes have strengthened the manuscript and hope it is now suitable for publication in PLOS Computational Biology.

Sincerely,

Katharina Eickhoff on behalf of all authors

Reviewer #1:

The authors propose a forward modeling approach allowing to combine the fine spatial resolution of fMRI and the precise temporal resolution from MEG measures. They predicted MEG responses at the brain level based on population receptive fields maps obtained from fMRI and visual stimuli presented during the MEG recordings. The predicted brain activity was then projected into the sensor space, and compared using ridge regression to the recorded MEG data. They claimed that the model was generalized across stimulus type and MEG sensor type. The visual cortex explains most of the variance of the model, compared to other visual areas. The encoding approach is methodologically sound and highly interesting, with strong potential to open new avenues for research. The manuscript is clearly written. However, it should be explicitly acknowledged that this work builds on a previous study by the authors,

which introduced the same modeling approach (Imaging Neuroscience, 2024) and the specific novelties and contributions of the present study should be highlighted. Some of the interpretations, particularly those concerning latency differences between brain areas and the generalizability of the model across stimuli, should be more cautiously framed, as they are not fully supported by the data. Last, I have also suggested additional points for discussion, including potential applications of the models and limitations related to stimulus generalizability.

Major points:

1. It should be clearly stated in the introduction that this modeling approach was already published earlier by the same authors (Eickhoff et al., 2024, Imaging Neuroscience, Population receptive field models capture the event-related magnetoencephalography response with millisecond resolution), and be highlighted what was done and what is the novelty of this study using the same dataset and approach.

We thank the reviewer for their constructive feedback and made changes to the manuscript based on the reviewer's suggestions.

We now clarify in the introduction that this modeling approach builds on our previous work and highlight its novelty. Previously, we focused on modeling data from individual MEG sensors using fMRI data from all visual regions; in contrast, our current approach leverages signals from all MEG sensors to capture activity from cortical patches, meaning we model the MEG data using specific cortical regions that were functionally defined based on the fMRI data. Ultimately, our goal is to understand brain function itself, with MEG sensors serving as a means to this end rather than being the primary focus.

Page 4 lines 91-95: "Building on our previous work that combines pRF models with fMRI and MEG [15,16], we now integrate signals across all MEG sensors to capture temporal information in specific cortical regions. Importantly, this approach allows us to characterize neural processing within brain regions using combined sensor data, rather than limiting our analysis to the sensor level."

*2. The predicted brain activity to MEG stimuli is a matrix (vertices*stimuli), according to Step 3 in the Method section. What is the unit of predicted activity, is it a binary matrix (1 if the vertex responds to the stimulus, 0 if it does not?)?*

The predicted brain activity is a continuous variable (with arbitrary units) that depends on the overlap of the pRF and the stimulus. We now clarify this in the text.

Page 5 lines 115-117: "<u>Third, we used the pRF models to predict responses to the same stimuli, such that activity of a given vertex scaled with the degree of overlap between the vertex' pRF model and the stimulus</u>."

On a similar note, how does the predicted responses at the sensor level look like? Please consider adding a topoplot and time course of predicted sensor activity to illustrate this point, for different stimuli for instance. It would be great also to have a plot with the individual evoked responses for each participant.

The predicted responses at the sensor level and topoplots are illustrated in our previous paper that the reviewer also mentions in a previous comment (Eickhoff et al, 2024, Imaging Neuroscience). The aim of the current work was to capture neural processing in brain regions by combining all sensors, rather than explaining measurements at the sensor or scalp level. We now elaborated on this logic more in the introduction (see first comment).

3. Generalization across stimulus type: by looking at the other 4 participants, it seems that the generalization is fine for the 200ms but quite bad for the rest of the tested temporal window. Different features of visual stimuli might impact the population receptive fields (see Altan et al., 2025, Spatial frequency adaptation modulates population receptive field sizes, for instance) and could explain a poor generalizability of the model. Here, the two tested stimuli (bars and circles) are quite similar (same spatial frequency) so we might expect even worst generalizability for very different stimuli. Wouldn't that be an important limitation to use this tool in other studies with hypothesis building on different stimuli? Or would you advise using this approach without generalizing across stimuli (i.e. the population receptive field should be calculated based on the same visual stimuli used during MEG recordings)?

As the reviewer correctly points out, neural processing depends on spatial and temporal stimulus properties and task demands. Likewise, pRF responses, and also single neuron RF, depend on stimulus layout.

Indeed, given these results, our recommendation for this approach is to keep the stimuli for fMRI and MEG as similar as possible.

That said, we do expect pRF models to generalise to some extent. The generalization across stimulus types explicitly tests the degree to which the pRF model and our approach generalizes across stimuli.

We now more explicitly discuss these issues in the Model generalizability and robustness section of the discussion.

Pages 11-12 lines 267-276: "Our approach generalized across stimuli, i.e., contrast-defined bar and circle stimuli (Fig 1C), although generalizability was somewhat lower across stimuli than sensor types. This is expected, as neural processing, including pRFs and single-neuron responses, varies with stimulus properties and task demands [43,43,44]. The larger bar stimuli, in particular, likely evoked more nonlinear responses such as compression, surround suppression, and oversaturation [45–48], which are not accounted for by our Gaussian model [17]. While advanced pRF models could improve generalizability [27,45,46,48], these findings also highlight the importance of aligning stimulus properties between fMRI and MEG. Nonetheless, our pRF model explained up to 66% of the response to a held-out stimulus, indicating it already provides a robust model of neural responses."

4. The authors should lower their claim about finding increasing latency along the visual hierarchy, as this was not significant. The exact statistics and outcomes are actually not detailed. To claim this, they should either use participant-level statistics or increase the number of participants in the group-level analysis.

We now provide new participant-level statistics to show that the activation windows are different along the visual hierarchy. Specifically, we determined whether V1 differs from extrastriate visual areas. This is the case for almost every other visual area in every participant. We show this analysis in a new figure panel (Figure 3C, pages 9-10 lines 209-222).

Pages 9-10 lines 209-222: "**Fig 3. Variance explained time-courses and activation timing for different visual field maps and clusters**. **A.** Median (solid) and 95% confidence intervals (shaded) for cross-validated variance explained for different visual field maps and clusters (rows) for participant 1 for 0 to 400 ms after stimulus onset (similar data as Fig 2A but with change of scale, see other participants in S3A Fig). The gray shaded area indicates stimulus presentation. The vertical ticks on the horizontal line below each variance explained time-course mark the time at which normalized cumulative variance explained reached 25, 50, and 75% of the total across the shown 400-ms time window. Note that the y-axes differ for each visual field map and cluster. **B**. 25, 50, 75% of the normalized cumulative variance explained time-course (S3B Fig), for all five participants (P1: solid line, P2: long dashed line, P3: short dashed line, P4: dash-dotted line, P5: dotted line). **C**. Latency of the 25% point. Distributions are shown as violin plots with the 95% confidence intervals. Significant differences between V1 and extrastriate latencies are indicated in opaque shading (Bonferroni-corrected $p<0.05$), whereas non-significant differences are shown in transparent shading."

Description in the results (page 10 lines 225-235): "To quantify the increasing latency from V1 to extrastriate cortex, we compared the latencies of the 25% points between V1 and extrastriate areas using a Fisher permutation test (10,000 permutations). These distributions are visualized as violin plots, reflecting the spread of latencies across 120 cross-validation folds. The white dot indicates the median onset across the 120 folds, and gray horizontal lines denote the 95% confidence interval of the latency distribution derived from these folds. We found that almost every extrastriate region in every participant had a significantly later latency than V1, except for V2 in P3 and V3 in P1 (Bonferroni-corrected $P<0.05$)."

Description of the analyses in the methods (pages 24-25 lines 579-592): "For the statistical analysis we performed the cross-validated fitting procedure 120 times using 120 random averages of the MEG data, referred to as cross-validation folds. We quantified activation onset for each visual field map and cluster (VFM) as the time point at which a fold's normalized cumulative cross-validated variance explained reached 25% of its total within the 0–400 ms post-stimulus window. For each participant and VFM, this procedure was applied to all 120 cross-validation folds, yielding a distribution of activation onset estimates per area.

We performed statistical comparisons of activation onset latencies within participants, comparing each extrastriate VFMs against V1. Differences were assessed using a paired Fisher permutation test based on 10,000 permutations, applied to the paired onset distributions across

folds. Resulting p-values were Bonferroni-corrected for multiple comparisons across visual field maps and clusters. Extrastriate areas with a corrected p-value < 0.05 were considered to show a significant latency difference relative to V1 and are indicated by opaque shading in the figure; non-significant comparisons are shown with transparent shading."

Minor points:

- Results section: when the authors mention the "visual field maps" it might be good to briefly precise how they were defined, as it was not clear (visual brain regions defined by hand based on the pRF analysis).

We now sharpened our description of how visual field maps and clusters were defined, and consistently refer to them as visual field maps and clusters.

Pages 18-19 lines 440-452: "We then defined visual field maps and clusters for each participant by hand (Fig 5A), based on the anatomical landmarks, and pRF polar angle and eccentricity estimates (Figs 5B-D). Visual field maps are defined as contiguous regions of cortex in which there exists a precise, systematic, and non-redundant mapping between locations in the visual field and specific locations on the cortical surface. In these regions, neighboring points in the visual field are represented by neighboring points on the cortex, preserving the topography and layout of visual space [21]. We identified four visual field maps: V1, V2, V3, hV4, and six visual field map clusters: V3ab, LO, VO, TO, posterior IPS (pIPS) and anterior IPS (aIPS). Visual field map *clusters* are spatially contiguous groups of visual field maps in the cortex that share common organizational features, such as a shared foveal representation. Note that we could have merged certain regions together (for example, posterior and anterior IPS), or separate them even further. However, for the purpose of this method, the current level of specificity matched other pRF modeling papers [25,45]."

- In the method section, the paragraph forward modeling approach/step 2 is a repetition of an earlier paragraph, please avoid redundancies.

We have now removed the repeating paragraph.

Page 19 lines 454-458: “We measured MEG data with 306 sensors at 102 sensor locations, while participants viewed different bar and circle stimuli (Fig 4C). At each location, the MEG system records three channels: one magnetometer measuring the absolute component of the magnetic field and two orthogonally oriented planar gradiometers measuring spatial gradients along two perpendicular tangential directions (Fig 1B).”

- “we identified ten visual field maps and clusters”: what do “clusters” refer to?

We now explicitly define the concept of clusters in the methods.

Page 19 lines 447-449: “Visual field map *clusters* are spatially contiguous groups of visual field maps in the cortex that share common organizational features, such as a shared foveal representation.”

- Fig S1B: what does that mean that the explained variance is negative?

This is cross-validated variance explained. Cross-validated variance explained can be negative if the model predicts worse than the mean on held-out data. An illustrative figure of this can also be found in the supplementary material of our previous paper Eickhoff et al, 2024, Imaging Neuroscience.

- The authors plotted twice the same data in Fig 2A and Fig 3A, with a change of scale. Although I understand the purpose, it might help to explicitly note that this is the same data.

We now explicitly mention this in the legend of Fig 3.

Page 9 lines 210-213: ”Median (solid) and 95% confidence intervals (shaded) for cross-validated variance explained for different visual field maps and clusters (rows) for participant 1 for 0 to 400 ms after stimulus onset (similar data as Fig 2A but with change of scale, see other participants in S3A Fig).”

- Can you give more details about the visual stimuli used in the MEG experiment? Is there a pattern reversal, or are they static?

We have updated our description of the MEG stimuli.

Page 16 lines 378-387: “The MEG stimuli were identical to those used by Eickhoff et al. [15] and comprised 18 contrast-defined apertures (Fig 4C): five vertical bars, five horizontal bars, and eight circles (overall stimulus extent 4.9°), yielding ~160 trials per stimulus. Bars were 1.25° wide and presented at 0°, 1.82°, or 3.06° eccentricity (five locations per orientation), whereas circles were presented in one of eight quadrant-specific locations (four quadrants × two eccentricities: 1.82° and 3.06°) with diameters of 1.25° and 2.5° at the small and large eccentricity, respectively. Stimuli were presented for 100 ms and followed by a mean-luminance screen (900 ms ± 250 ms) to evoke event-related fields (ERFs); during the 100 ms presentation, the aperture remained static while the checkerboard rows moved in opposite directions. Participants performed a fixation color-change task throughout.”

- The authors might consider expending on how their approach might be used, for which application, or to test which kind of research question.

We now expand on this in the conclusion:

Page 14 lines 336-345 :“Our approach estimates how individual cortical regions contribute to visual responses in humans. By combining fMRI and MEG with biologically-inspired models, we achieve temporal resolutions comparable to invasive electrophysiology. Our forward modeling relies on biologically inspired pRF models [17]. Beyond bridging fMRI and MEG, these models offer interpretable, mechanistic insights into brain function [34,35]. They have already expanded from vision to sensory [67,68], cognitive [9,69,70], and clinical domains, including ophthalmological, neurological, and psychiatric conditions [71–73]. Within the pRF framework, the proposed approach allows us to explore the timing of neural computations across these different domains and conditions. More broadly, it can be adapted to any biologically inspired computational model.”

- To the editors/authors: please consider adding page and line numbers to ease the review.

Yes.

Reviewer #2

This manuscript proposes a forward modeling method integrating high spatial resolutional fMRI and high temporal resolution MEG to map the temporal processing hierarchy in the human visual cortex.

While this work addresses an interesting methodological topic, I find that its theoretical novelty and practical significance are significantly limited. Therefore, I do not recommend it for publication in its current form. Below, I detial my primary concerns:

Major concerns:

(1) Although the introduction emphasizes the importance of integrating spatial an temporal information, it lacks sufficient theoretical motivation or clear hypotheses regarding the necessity of detailed subdivision of visual field maps or integration based specifically on pRF. Without a strong theoretical framework, the study's contribution remains unclear.

We thank the reviewer for their constructive comments and we have changed the manuscript accordingly.

Vision, and brain processing more broadly, is inherently dynamic across space and time, so understanding brain function requires considering both spatial and temporal dimensions. Regarding spatial dimensions, a cardinal feature of the visual system is its subdivision into visual fields maps and its finer functional architecture based on receptive fields. These can now be studied noninvasively using fMRI and pRF models, but this unimodal approach cannot yield full understanding of what neural processing goes on within functional brain regions. To this end a finer temporal resolution is required. There is no noninvasive recording technique available that provides both temporal and spatial precision. Hence, we set out to combine two recording techniques, fMRI providing spatial precision and MEG providing temporal precision. We believe such a combination of recording techniques is needed to take the next step towards understanding visual processing. We provide a new method to estimate neural processing at both high temporal and spatial resolution based on biologically inspired models in the living human brain.

In the abstract we sharpen this rationale (page 2 lines 27-29): "Vision, and brain processing more broadly, is inherently dynamic across space and time, so understanding brain function requires consideration of both spatial and temporal dimensions."

We also now refine the practical significance and theoretical framework of our work in the introduction (page 4 line 91 onwards): “Building on our previous work that combines pRF models with fMRI and MEG [15,16], we now integrate signals across all MEG sensors to capture temporal information in specific cortical regions. Importantly, this approach allows us to characterize neural processing within brain regions using combined sensor data, rather than limiting our analysis to the sensor level. Biologically-inspired models, such as the pRF model, are valuable because they offer interpretable, mechanistic insights into how the brain processes information, linking neural activity to specific stimulus features [...]”

(2) Representational similarity analysis (RSA) is a common and well-established method for fMRI-E/MEG fusion analysis. The manuscript does not compare its proposed method with RSA, nor does it discuss RSA approaches in either the Intro or Discussion. Given RSA's widespread use and relevance, this omission significantly weakens the manuscript.

The goal of this work was not only to fuse fMRI and MEG, but to do so with mechanistic models of brain processing, in our case the pRF model, which RSA would not provide.

We now also refer to representational similarity analysis (RSA) in the introduction and sharpened our rationale for using biologically inspired models.

Pages 4-5 lines 95-99: “Biologically-inspired models, such as the pRF model, are valuable because they offer interpretable, mechanistic insights into how the brain processes information, linking neural activity to specific stimulus features, whereas methods like representational similarity analysis (RSA) focus primarily on similarities in activation patterns without tying them directly to underlying neural computations or properties.”

(3) The methodological approach appears limited to specific stimulus paradigms (e.g., pRF-type stimuli). It is unclear if this method can generalize to other experimental conditions or stimuli, restricting its broader applicability.

Our approach can be adapted to any biologically inspired computational model and we now explicitly discuss this broader applicability in the discussion.

Page 14 lines 336-345: "Our approach estimates how individual cortical regions contribute to visual responses in humans. By combining fMRI and MEG with biologically-inspired models, we achieve temporal resolutions comparable to invasive electrophysiology. Our forward modeling relies on biologically inspired pRF models [17]. Beyond bridging fMRI and MEG, these models offer interpretable, mechanistic insights into brain function [34,35]. They have already expanded from vision to sensory [67,68], cognitive [9,69,70], and clinical domains, including ophthalmological, neurological, and psychiatric conditions [71–73]. Within the pRF framework, the proposed approach allows us to explore the timing of neural computations across these different domains and conditions. More broadly, it can be adapted to any biologically inspired computational model."

(4) The hierarchical processing results obtained here are not novel. This study doesn't substantially advance our theoretical understanding or provide meaningful new insights.

The novelty is not in the temporal processing results but the ability to estimate the processing hierarchy non-invasively in the living human brain through computational models in combination with multimodal imaging.

We now state this more explicitly in the first paragraph of the discussion.

Pages 10-11 lines 241-245: "Our hierarchical processing results are consistent with invasive electrophysiology in both non-human primates and humans [2,25,26] and recent fMRI modeling [27]. However, compared to these previous approaches, our method uniquely provides non-invasive measures in humans that have high spatio-temporal resolution measures as well as full brain coverage."

Other concerns:

*(1) In Fig 1, the subfigure labeled "ERFs_t (sensors * stimuli)" appears incorrectly represented. Since it refers to MEG signals at a specific timepoint (t), it should not contain an additional temporal dimension.*

As the reviewer correctly points out, the MEG signal refers to a specific timepoint (t), so we removed the "_t" and replaced it with "(t)" (page 6 Fig 1).

(2) Onset latency should also be analyzed and reported alongside peak latency for a comprehensive view of temporal dynamics.

We performed new participant-level statistics to show latency differences across the visual hierarchy, specifically we determined whether V1 differs from extrastriate visual areas. For this we analyzed the onset as the 25% point of the cumulative time-courses, to take into account the variability between regions and participants in the number and timing of peaks in the raw time-courses (see also S4 Fig).

The report of this latency analysis can be found in the answers to Reviewer #1, or on these pages in the manuscript: new figure and description on pages 9-10 lines 209-222, report of findings in results on page 10 lines 225-235, and detailed description of analysis on pages 24-25 lines 579-592.

(3) The impact of varying numbers of repeated trials on the robustness of results is not explored, which is essential for guiding future experimental designs.

In the current study the validity of the number of repeated trials is demonstrated by the generalizability of our findings across stimulus types, sensor types, and numbers of regressors.

For future experimental designs with similar stimulus and task properties a similar or smaller number of repeated trials may be adopted, but given the robustness of our results, we anticipate that fewer repetitions of stimuli could still yield accurate fits while requiring less time for the participant in the scanner. The number of repeated trials may be further reduced when the performed analyses do not involve data splitting and cross-validation.

The number of repetitions required will ultimately depend on the signal-to-noise ratio of the neural responses, which is shaped by both stimulus characteristics and task demands, with noisier responses needing more repetitions to obtain reliable estimates.

(4) Similarly, discussion on the minimal number of trials or stimulus conditions required for reliable generalization is lacking. Clearly outlining these basic experimental requirements would be highly beneficial for future users of this method.

We now discuss this issue explicitly in the first paragraph of the limitations section.

Pages 13-14 lines 310-319: "While existing atlases may be used to reduce this burden [54,56,57], future experimental designs may also benefit from optimizing the number of stimulus repetitions. In the current study, the robustness and generalizability of our results across sensor types, stimulus types and number of regressors, suggests that a comparable or smaller number of repetitions may be sufficient for experiments with similar stimulus and task properties. In particular, designs that do not require data splitting and cross-validation may allow for fewer repetitions, reducing participant time in the scanner. Ultimately, the number of repetitions required will depend on the signal-to-noise ratio of the neural responses, which is shaped by stimulus characteristics and task demands of the experiment [58,59]."

Reviewer #3

This paper describes a technique that estimates the temporal evolution of activity in the visual cortex. The technique uses fMRI to estimate visual field maps and then employs a forward model to predict the MEG response. This prediction is done across time, such that each field map has an estimated temporal response accuracy for predicting the MEG response.

Overall assessment:

This paper provides a powerful tool to estimate of the contribution of each field map to visual responses over time. The technical aspects are solid, rigorous cross-validation is used to show that the model can explain responses to left out stimuli and the paper is clearly written. To further strengthen the paper, it would be particularly helpful to show the degree to which the proposed approach can replicate known temporal dynamics of the visual system. Moreover, there are some methodological and conceptual issues that need to be addressed.

Major comments:

1) Can the proposed method reproduce known temporal dynamics in the visual system? Previous intracranial studies measured onset latencies in the visual stream in humans (e.g. Martin et al., 2019, JNeurosci, Temporal Dynamics and Response Modulation across the Human Visual System in a Spatial Attention Task: An ECoG Study and Groen et al., 2022, JNeurosci, Temporal Dynamics of Neural Responses in Human Visual Cortex). Both these

studies found that IPS has a particularly early response onset. Can the proposed model reproduce this finding?

We thank the reviewer for their constructive comments.

We reproduce the general trend of temporal dynamics observed in the human visual system, with lower-level region V1 generally activating earlier than higher-level regions.

Regarding IPS, we here split IPS in two subsections. It is known that IPS consists of many different regions with many different properties. Our method potentially allows us to estimate the contribution of each of these areas at the expense of increasing the number of regressors.

In the intracranial studies mentioned by the reviewer the individual electrodes covered only a small part of IPS, likely capturing signals from a limited number of IPS subregions. In Fig 3A and S2 Fig early activations of our bigger subsections of IPS (pIPS and aIPS) are visible in some but not all participants. This may be due to individual variation, or a consequence of the rather large subsections we defined, which combine many IPS subregions and hence merges their contributions. Because of the limited number of participants, and substantial variation between participants, we refrain from detailed comparisons. The focus of this paper is to establish the method, yet the reviewer's question is an interesting topic for future studies.

2) What could be an explanation be for the delayed responses in V2 compared to V3 in Figure 3A and 3B?

Because of the limited number of participants and the substantial variation between participants (see S2 Fig), we refrain from detailed comparisons between V2 and V3. Hence, it remains unknown whether there is a significant difference between V2 and V3 latency in this participant. Generally, multiple factors may underlie a difference between any two regions. It may be a true difference or it may be due to other factors like a correlation between the signals generated by the two regions.

3) The results and conclusion confuse measured responses and estimates of contributions. The contributions were not directly measured. (e.g. first sentence of the results: "To measure the time-courses of visual field maps..." and "We non-invasively measured the contribution…")

We have corrected these sentences and others to sharpen which results reflect estimates and which measurements.

Page 2 lines 32-33: "[...] enabling us to non-invasively estimate different levels of the visual hierarchy [...]"

Page 5 line 111: "To estimate the time-courses of [...]"

Page 6 line 125: "We estimated each participant's population receptive fields (pRFs) [...]"

4) Several studies have shown that evoked potentials, as measured here with MEG, are not directly related to the BOLD response measured with fMRI. In visual cortex, for example, it has been shown that these two measurements integrate differently across space (e.g. Winawer et al., 2013, Current Biology, Asynchronous Broadband Signals Are the Principal Source of the BOLD Response in Human Visual Cortex), with different non-linearities. Other studies comparing MEG and fMRI further showed that different neural response types result in different predicted spatial distributions (e.g. Kupers et al., 2021, A visual encoding model links magnetoencephalography signals to neural synchrony in human cortex). It should be discussed how such different differences in signals affect/are accounted for in the proposed method.

Indeed, fMRI and MEG have different sensitivities to neural activity, as is evident from the examples that the reviewer provides as well as other studies that we now reference in the manuscript (see below). Still, they are largely by the same neural activations and our method utilizes this commonality between the signals.

We now elaborate on the different sensitivities of fMRI and MEG to neural signals in the discussion.

Page 14 lines 320-327: "fMRI and MEG measure different neural signals: fMRI detects changes in blood oxygenation related to neural metabolism, while MEG records the magnetic fields generated by synchronized neural electrical activity. These differences ultimately limit the fusion of fMRI and MEG data. For example, fMRI may fail to detect neural activity beneath large draining veins due to vascular artifacts, whereas MEG can still capture the underlying signals [58]. Conversely, MEG may miss desynchronized activity because such patterns produce little net magnetic field, while fMRI can detect the associated metabolic changes [59].

Yet despite these differences, MRI-based pRFs explain a large portion of the MEG signals [15,16]."

5) More background should be provided for testing and training across the magnetometers and planar gradiometers, as it is unclear to the reader whether these different sensors may emphasize different types of neural responses and whether these measurements would be expected to match or mismatch?

We now explicitly explain the rationale in the discussion.

Page 11 lines 258-266: "Our approach generalized across MEG sensors, i.e., magnetometers and planar gradiometers (Fig 1B), which differ in what they measure. Magnetometers detect the magnetic field at a point and are sensitive to both brain signals and distant noise. The planar gradiometers, on the other hand, measure the magnetic field gradient between two nearby points in two orthogonal directions on the sensor plane. This makes them more sensitive to local brain activity directly beneath the sensor and less affected by distant interference. Importantly, the underlying neural sources of the recorded fields/field gradients are the same for the two sensor types. The ability to generalize across these different types of sensors, therefore, highlights the capacity of the pRF model to capture sensor-independent neural activity."

We also describe them in the methods.

Page 19 lines 454-458: "We measured MEG data with 306 sensors at 102 sensor locations, while participants viewed different bar and circle stimuli (Fig 4C). At each location, the MEG system records three channels: one magnetometer measuring the absolute component of the magnetic field and two orthogonally oriented planar gradiometers measuring spatial gradients along two perpendicular tangential directions (Fig 1B)."

Minor:

The methods should state which degree of visual angle was spanned by the stimuli (assuming 5.34 degrees diameter based on Figure 5?).

We have updated our description of the MEG stimuli including the degree of visual angle.

Page 16 lines 378-384: "The MEG stimuli were identical to those used by Eickhoff et al. [15] and comprised 18 contrast-defined apertures (Fig 4C): five vertical bars, five horizontal bars, and eight circles (overall stimulus extent 4.9°), yielding ~160 trials per stimulus. Bars were 1.25° wide and presented at 0°, 1.82°, or 3.06° eccentricity (five locations per orientation), whereas circles were presented in one of eight quadrant-specific locations (four quadrants × two eccentricities: 1.82° and 3.06°) with diameters of 1.25° and 2.5° at the small and large eccentricity, respectively."

It is unclear in the Figure 1 caption that r = 0.9-1.0 is meant to indicates a min-max range.

We now explicitly mention this.

Page 6 lines 143-146: "The time-courses were highly similar, indicating that V1's time-course estimates were robust to the number of extrastriate visual field maps and clusters included in the model (minimum and maximum $r(398) = 0.90$ and 1.0, $p < 0.001$)."

In the introduction "As a true model of neuronal activity" the term 'true' seems overstated.

We removed the term and restructured the introduction.

Page 4 line 91-97: "Building on our previous work that combines pRF models with fMRI and MEG [15,16], we now integrate signals across all MEG sensors to capture temporal information in specific cortical regions. Importantly, this approach allows us to characterize neural processing within brain regions using combined sensor data, rather than limiting our analysis to the sensor level. Biologically-inspired models, such as the pRF model, are valuable because they offer interpretable, mechanistic insights into how the brain processes information, linking neural activity to specific stimulus features, [...]"

In the method sentence: "Before the data matrices were fed into the ridge regression, the datapoints across all sensor and stimuli" sensor should be plural.

Corrected (page 21 line 500).

Once again, thank you very much for your time.

Sincerely,

Katharina Eickhoff, and all other authors
