# Supplementary material for: Non-invasive mapping of the temporal processing hierarchy in the human visual cortex
Source: PLoS Comput Biol. 2026 Jul 10;22(7):e1014434. doi: 10.1371/journal.pcbi.1014434 (PMC13379088; doi:10.1371/journal.pcbi.1014434)
Supplement: Attachment — Submitted filename: Response_to_Reviewers_auresp_2.pdf [file pcbi.1014434.s010.pdf]

Response to reviewers

Dear Yuanning Li and Hugues Berry,

We thank reviewers 2 and 3 for indicating that their concerns have been addressed, and we thank reviewer 1 for the additional careful reading of the revised manuscript. We have further revised the paper in response. Below we provide a point-by-point response, with reviewer comments reproduced in *green italics* followed by our responses. These revisions have, in our view, further improved the clarity and precision of the manuscript, and we hope it is now suitable for publication in *PLOS Computational Biology*.

Sincerely,

Katharina Eickhoff on behalf of all authors

Reviewer #1:

The authors have addressed most of my previous comments, but several important issues remain. Although they now provide solid statistical evidence showing that V1 responses occur earlier than those in other visual regions, the data do not fully support claims of a hierarchical progression across extrastriate areas, as latency increases along the visual stream were not directly tested. In addition, the use of tSSS preprocessing renders magnetometer and gradiometer signals non-independent, which limits the relevance of assessing model robustness across sensor types. The manuscript also does not provide guidance regarding the number of participants required to draw reliable conclusions, an aspect that would be particularly valuable in a methodological paper. Finally, the discussion still lacks a clear and specific articulation of the unique questions enabled by the proposed approach, a point previously raised by all reviewers.

Major points:

1. The preprocessing of the MEG data should be described in sufficient detail in the present manuscript so that readers do not need to refer to the first article using this dataset. It appears that temporal Signal Space Separation (tSSS) was applied, as is common in MEG preprocessing. However, this procedure introduces signal mixing between magnetometers and gradiometers (e.g., Garcés et al., 2017, Sensors, Choice of Magnetometers and Gradiometers after Signal Space Separation). As a result, assessing the robustness of the model across sensor types is of limited interest, since the signals are no longer independent. This point should at least be discussed.

We agree with the reviewer on both points.

First, the preprocessing should be sufficiently described in the present manuscript, and we have revised the Methods accordingly so that the reader does not need to consult the earlier paper for essential preprocessing details:

> "The MEG preprocessing followed the pipeline described in Eickhoff et al. [15], but we summarize the essential steps here. MEG data were acquired at 1000 Hz with an online high-pass filter at 0.10 Hz and low-pass filter at 330 Hz, using internal active shielding. Raw data were first preprocessed with temporal signal space separation (tSSS [77]; MaxFilter Software Elekta Neuromag, Oy, version 2.2.15) to reduce environmental noise and compensate for head movement; malfunctioning or noisy channels were interpolated during this step (mean 5.5 of 306 channels). Stimulus onsets were identified using a photodiode recorded alongside the MEG data, and periods containing missed screen flips were discarded. Data were then epoched from -100 to 600 ms relative to stimulus onset, yielding on average 186.2 epochs per stimulus, and each epoch was baseline corrected using the -100 to 0 ms prestimulus interval. No further preprocessing or filtering steps were applied before computing event-related fields (ERFs) used in the modeling analyses, however, subsequent analyses were restricted to the 0 to 400 ms post-stimulus window." (page 17, lines 404-416)

Second, we agree that tSSS reduces the statistical independence between magnetometer and gradiometer signals by projecting them into a shared signal space, as discussed by Garcés et al. (2017). More broadly, these sensor types are not fully independent even before tSSS, because both reflect the same underlying neural generators, albeit through different measurement profiles. Our intent in this validation was therefore not to claim independence between sensor types, but to test whether the model generalizes across different sensor representations of the same underlying neural activity.

To make this explicit, we have revised the Discussion to clarify both the limitation and the interpretation of this validation analysis. The manuscript now states:

> "Although signal space separation preprocessing reduces the statistical independence between sensor types [64], it is still valuable to assess whether our model generalizes across different sensor representations of the same underlying neural activity. The fact that performance was preserved across sensor types suggests that the model captures features of the underlying signal rather than sensor-specific characteristics." (page 14, lines 337-342)

2. The authors only tested whether the latency in extrastriate regions are different from V1 latency, but they did not test whether there was an increase in latency along visual regions. They cannot claim to report "a processing hierarchy across extrastriate visual fields maps" (p2 l45).

We agree and have removed this sentence. The revised manuscript now restricts this conclusion to the distinction between striate (V1) and extrastriate cortex.

3. The authors claim that their method enables the identification of a temporal hierarchy in visual processing. However, when Reviewer 3 requested clarification regarding the observed delay between V2 and V3 responses or asked whether a known finding in the literature might be replicated (early IPS onset), the authors argued that the sample size was too small to draw firm conclusions. This raises an important question: how many participants would be required to reliably detect such differences? Providing at least an estimate or power analysis would be

particularly valuable in a methodological paper, as it would help guide future users regarding the expected sensitivity and practical requirements of the approach.

While we agree that guidance on the number of participants required is important, particularly for a methodological paper, at the same time, we do not think that a single general recommendation can be derived from the present dataset.

The key reason is that the statistical framework used here operates at the within-participant level, where sensitivity is driven not only by the number of participants, but also by the number of trials per participant, the signal-to-noise ratio of the responses, the stimulus design, and the analysis choices (e.g., cross-validation, model complexity, and regularization). These factors jointly determine statistical power and are likely to vary substantially across future applications of the method.

In the present study, the data were sufficient to robustly detect differences between V1 and extrastriate cortex at the individual level, but not to resolve more subtle differences between extrastriate areas across participants. We have revised the manuscript to make this distinction explicit and to frame the present work as a validation of the method rather than as a study designed to establish general sample-size requirements.

> "More generally, statistical sensitivity in this framework depends not only on participant number, but also on the number of trials per participant, the signal-to-noise ratio, the stimulus design, and the analysis choices. In the present dataset, these factors were sufficient to detect V1 versus extrastriate latency differences, but not more subtle differences among extrastriate areas. Future studies targeting such finer-grained effects will likely require more data, either through additional participants, more trials, or both." (pages 13-14, lines 317-322)

4. Finally, in line with the two other reviewers' comments, I agree that the manuscript would benefit from a more explicit discussion of how the proposed method enables the field to address specific questions that could not be answered with previous approaches. Clearly articulating these added-value aspects would substantially strengthen the paper, as the current discussion remains too general.

We have revised the Discussion and Conclusion to articulate this more explicitly.

> "Our approach estimates how individual cortical regions contribute to visual responses in humans. By combining fMRI and MEG with biologically-inspired models, we achieve temporal resolutions comparable to invasive electrophysiology while retaining the spatial specificity of functionally defined visual field maps. Our forward modeling relies on biologically inspired pRF models [17], which provide mechanistic insights into brain function [34,35]. Beyond bridging fMRI and MEG, the proposed approach enables us to pinpoint when specific cortical regions contribute to visual processing in individual humans, and how that timing changes with stimulus properties, task demands, or disease. Building on the successful extension of pRF models from vision to sensory [70,71], cognitive [9,72,73], and clinical domains, including ophthalmological, neurological, and psychiatric conditions [74–76], this framework opens new opportunities to study the timing of neural computations across these different domains and conditions. More broadly, it can be adapted to any biologically inspired computational model." (page 15, lines 349-361)

Minor points:

- One of my previous points has not been fully addressed. I had asked for a figure showing the predicted sensor-level activity associated with the model's predicted responses, so that readers can directly appreciate what these predictions look like. The authors indicate that such a plot is available in their previous article (Eickhoff et al., 2024, Imaging Neuroscience). However, that article does not include predictions derived from visual field maps, which constitute the novelty of the present study.

I still believe it would be highly informative to visualize these predictions within a classical ERP framework, as this would allow readers to assess which components align more closely with the different visual field map–based predictions.

We thank the reviewer for this suggestion. However, the primary aim of the present study is to estimate the temporal contribution of functionally defined cortical regions by combining

information across all MEG sensors, rather than to interpret responses at the level of individual sensors or classical ERP components.

Thus, including detailed sensor-level visualizations would shift the focus back to sensor space and detract from the central methodological advance of the paper.

We therefore chose not to add such a figure in the present manuscript. To ensure that readers can still appreciate how model predictions relate to MEG measurements, we refer to our previous work (Eickhoff et al., 2024), where predicted and measured sensor-level responses are visualized.

- One limitation of the model that would be worth discussing is that it only considers static stimuli (as in the prediction stage, a single snapshot of the presented stimuli is used). How could you adapt your model to dynamically evolving stimuli (for instance a moving bar)?

In the present implementation, the model predicts responses to briefly presented, spatially defined apertures and therefore uses a static description of the stimulus at each event. For dynamically evolving stimuli, the same framework could in principle be extended by replacing the static stimulus representation with a time-resolved stimulus sequence and generating time-varying model predictions at the cortical level before projection to MEG sensor space. In that setting, the encoding model would need to capture not only spatial selectivity but also temporal dynamics, and potentially motion-dependent nonlinearities. We now note this explicitly as an important direction for future work.

In the Discussion:

> "In its current implementation, the model is tailored to briefly presented static apertures; extending it to dynamic stimuli, such as a moving bar, would require generating time-resolved predictions that track the stimulus over time." (page14, lines 324-327)

- In Figure 3C, it is not possible to clearly distinguish which violin plots are shown as opaque or transparent (indicating whether latencies differ significantly from V1).

To improve clarity, we have revised the figure to indicate non-significance explicitly (with “n.s.”), rather than relying only on opacity differences.

Once again, thank you very much for your time and effort.

Sincerely,

Katharina Eickhoff, and all other authors
