# Supplementary material for: Non-invasive mapping of the temporal processing hierarchy in the human visual cortex
Source: PLoS Comput Biol. 2026 Jul 10;22(7):e1014434. doi: 10.1371/journal.pcbi.1014434 (PMC13379088; doi:10.1371/journal.pcbi.1014434)
Supplement: Peer review history

# Peer review: Decision Letter 0

**DOI:** 10.1371/journal.pcbi.1014434.r001

**Authors:** Hugues Berry, Yuanning Li

7 Aug 2025

PCOMPBIOL-D-25-01036

Non-invasive mapping of the temporal processing hierarchy in the human visual cortex

PLOS Computational Biology

Dear Dr. Eickhoff,

Thank you for submitting your manuscript to PLOS Computational Biology. After careful consideration, we feel that it has merit but does not fully meet PLOS Computational Biology's publication criteria as it currently stands. Therefore, we invite you to submit a revised version of the manuscript that addresses the points raised during the review process.

Please submit your revised manuscript within 60 days Oct 07 2025 11:59PM. If you will need more time than this to complete your revisions, please reply to this message or contact the journal office at ploscompbiol@plos.org. When you're ready to submit your revision, log on to https://www.editorialmanager.com/pcompbiol/ and select the 'Submissions Needing Revision' folder to locate your manuscript file.

Please include the following items when submitting your revised manuscript:

* A rebuttal letter that responds to each point raised by the editor and reviewer(s). You should upload this letter as a separate file labeled 'Response to Reviewers'. This file does not need to include responses to formatting updates and technical items listed in the 'Journal Requirements' section below.

* A marked-up copy of your manuscript that highlights changes made to the original version. You should upload this as a separate file labeled 'Revised Manuscript with Track Changes'.

* An unmarked version of your revised paper without tracked changes. You should upload this as a separate file labeled 'Manuscript'.

If you would like to make changes to your financial disclosure, competing interests statement, or data availability statement, please make these updates within the submission form at the time of resubmission. Guidelines for resubmitting your figure files are available below the reviewer comments at the end of this letter

We look forward to receiving your revised manuscript.

Kind regards,

Yuanning Li

Academic Editor

PLOS Computational Biology

Hugues Berry

Section Editor

PLOS Computational Biology

**Journal Requirements:**

1) We note that your Supplementary Figures files are duplicated on your submission as they are included in the manuscript and uploaded as separate files. Please remove the Supplementary Figures from the main file of the manuscript as they should be uploaded as separate files.

2) We notice that your supplementary figures are uploaded with the file type 'Figure'. Please amend the file type to 'Supporting Information'. Please ensure that each Supporting Information file has a legend listed in the manuscript after the references list.

3) We ask that a manuscript source file is provided at Revision. Please upload your manuscript file as a .doc, .docx, .rtf or .tex. If you are providing a .tex file, please upload it under the item type u2018LaTeX Source Fileu2019 and leave your .pdf version as the item type u2018Manuscriptu2019.

4) Some material included in your submission may be copyrighted. According to PLOSu2019s copyright policy, authors who use figures or other material (e.g., graphics, clipart, maps) from another author or copyright holder must demonstrate or obtain permission to publish this material under the Creative Commons Attribution 4.0 International (CC BY 4.0) License used by PLOS journals. Please closely review the details of PLOSu2019s copyright requirements here: PLOS Licenses and Copyright. If you need to request permissions from a copyright holder, you may use PLOS's Copyright Content Permission form.

Please respond directly to this email and provide any known details concerning your material's license terms and permissions required for reuse, even if you have not yet obtained copyright permissions or are unsure of your material's copyright compatibility. Once you have responded and addressed all other outstanding technical requirements, you may resubmit your manuscript within Editorial Manager.

Potential Copyright Issues:

i) Figures 1A, and 5. Please confirm whether you drew the images / clip-art within the figure panels by hand. If you did not draw the images, please provide (a) a link to the source of the images or icons and their license / terms of use; or (b) written permission from the copyright holder to publish the images or icons under our CC BY 4.0 license. Alternatively, you may replace the images with open source alternatives. See these open source resources you may use to replace images / clip-art:

- https://commons.wikimedia.org

- https://openclipart.org/.

5) Thank you for stating that " Analysis code will be made publicly available on GitHub." Please note that, though access restrictions are acceptable now, your entire data will need to be made freely accessible if your manuscript is accepted for publication. This policy applies to all data except where public deposition would breach compliance with the protocol approved by your research ethics board.

6) In the online submission form, you indicated that "Minimally preprocessed data will be made available upon request."  All PLOS journals now require all data underlying the findings described in their manuscript to be freely available to other researchers, either

1. In a public repository

2. Within the manuscript itself

3. Uploaded as supplementary information.

PLOS only allows data to be available upon request if there are legal or ethical restrictions on sharing data publicly. For more information on unacceptable data access restrictions, please see https://journals.plos.org/ploscompbiol/s/data-availability#loc-unacceptable-data-access-restrictions.

Before we proceed with your manuscript, please address the following prompts:

a) If there are ethical or legal restrictions on sharing a de-identified data set, please explain them in detail (e.g., data contain potentially identifying or sensitive patient information, data are owned by a third-party organization, etc.) and who has imposed them (e.g., a Research Ethics Committee or Institutional Review Board, etc.). Please also provide contact information for a data access committee, ethics committee, or other institutional body to which data requests may be sent.

b) If there are no restrictions, please upload the minimal anonymized data set necessary to replicate your study findings to a stable, public repository and provide us with the relevant URLs, DOIs, or accession numbers. For a list of recommended repositories, please see https://journals.plos.org/plosone/s/recommended-repositories. You also have the option of uploading the data as Supporting Information files, but we would recommend depositing data directly to a data repository if possible.

7) Please ensure that the grant recipient is included in the Funding Information tab.

Note: If the reviewer comments include a recommendation to cite specific previously published works, please review and evaluate these publications to determine whether they are relevant and should be cited. There is no requirement to cite these works unless the editor has indicated otherwise.

**Reviewers' comments:**

Reviewer's Responses to Questions

Reviewer #1: The authors propose a forward modeling approach allowing to combine the fine spatial resolution of fMRI and the precise temporal resolution from MEG measures. They predicted MEG responses at the brain level based on population receptive fields maps obtained from fMRI and visual stimuli presented during the MEG recordings. The predicted brain activity was then projected into the sensor space, and compared using ridge regression to the recorded MEG data. They claimed that the model was generalized across stimulus type and MEG sensor type. The visual cortex explains most of the variance of the model, compared to other visual areas. The encoding approach is methodologically sound and highly interesting, with strong potential to open new avenues for research. The manuscript is clearly written. However, it should be explicitly acknowledged that this work builds on a previous study by the authors, which introduced the same modeling approach (Imaging Neuroscience, 2024) and the specific novelties and contributions of the present study should be highlighted. Some of the interpretations, particularly those concerning latency differences between brain areas and the generalizability of the model across stimuli, should be more cautiously framed, as they are not fully supported by the data. Last, I have also suggested additional points for discussion, including potential applications of the models and limitations related to stimulus generalizability.

Major points:

1. It should be clearly stated in the introduction that this modeling approach was already published earlier by the same authors (Eickhoff et al., 2024, Imaging Neuroscience, Population receptive field models capture the event-related magnetoencephalography response with millisecond resolution), and be highlighted what was done and what is the novelty of this study using the same dataset and approach.

2. The predicted brain activity to MEG stimuli is a matrix (vertices*stimuli), according to Step 3 in the Method section. What is the unit of predicted activity, is it a binary matrix (1 if the vertex responds to the stimulus, 0 if it does not?)? On a similar note, how does the predicted responses at the sensor level look like? Please consider adding a topoplot and time course of predicted sensor activity to illustrate this point, for different stimuli for instance. It would be great also to have a plot with the individual evoked responses for each participant.

3. Generalization across stimulus type: by looking at the other 4 participants, it seems that the generalization is fine for the 200ms but quite bad for the rest of the tested temporal window. Different features of visual stimuli might impact the population receptive fields (see Altan et al., 2025, Spatial frequency adaptation modulates population receptive field sizes, for instance) and could explain a poor generalizability of the model. Here, the two tested stimuli (bars and circles) are quite similar (same spatial frequency) so we might expect even worst generalizability for very different stimuli. Wouldn’t that be an important limitation to use this tool in other studies with hypothesis building on different stimuli? Or would you advise using this approach without generalizing across stimuli (i.e. the population receptive field should be calculated based on the same visual stimuli used during MEG recordings)?

4. The authors should lower their claim about finding increasing latency along the visual hierarchy, as this was not significant. The exact statistics and outcomes are actually not detailed. To claim this, they should either use participant-level statistics or increase the number of participants in the group-level analysis.

Minor points:

- Results section: when the authors mention the “visual field maps” it might be good to briefly precise how they were defined, as it was not clear (visual brain regions defined by hand based on the pRF analysis).

- In the method section, the paragraph forward modeling approach/step 2 is a repetition of an earlier paragraph, please avoid redundancies.

- “we identified ten visual field maps and clusters”: what do “clusters” refer to?

- Fig S1B: what does that mean that the explained variance is negative?

- The authors plotted twice the same data in Fig 2A and Fig 3A, with a change of scale. Although I understand the purpose, it might help to explicitly note that this is the same data.

- Can you give more details about the visual stimuli used in the MEG experiment? Is there a pattern reversal, or are they static?

- The authors might consider expending on how their approach might be used, for which application, or to test which kind of research question.

- To the editors/authors: please consider adding page and line numbers to ease the review.

Reviewer #2: This manuscript proposes a forward modeling method integrating high spatial resolutional fMRI and high temporal resolution MEG to map the temporal processing hierarchy in the human visual cortex.

While this work addresses an interesting methodological topic, I find that its theoretical novelty and practical significance are significantly limited. Therefore, I do not recommend it for publication in its current form. Below, I detial my primary concerns:

Major concerns:

(1) Although the introduction emphasizes the importance of integrating spatial an temporal information, it lacks sufficient theoretical motivation or clear hypotheses regarding the necessity of detailed subdivision of visual field maps or integration based specifically on pRF. Without a strong theoretical framework, the study's contribution remains unclear.

(2) Representational similarity analysis (RSA) is a common and well-established method for fMRI-E/MEG fusion analysis. The manuscript does not compare its proposed method with RSA, nor does it discuss RSA approaches in either the Intro or Discussion. Given RSA's widespread use and relevance, this omission significantly weakens the manuscript.

(3) The methodological approach appears limited to specific stimulus paradigms (e.g., pRF-type stimuli). It is unclear if this method can generalize to other experimental conditions or stimuli, restricting its broader applicability.

(4) The hierarchical processing results obtained here are not novel. This study doesn't substantially advance our theoretical understanding or provide meaningful new insights.

Other concerns:

(1) In Fig 1, the subfigure labeled “ERFs_t (sensors * stimuli)” appears incorrectly represented. Since it refers to MEG signals at a specific timepoint (t), it should not contain an additional temporal dimension.

(2) Onset latency should also be analyzed and reported alongside peak latency for a comprehensive view of temporal dynamics.

(3) The impact of varying numbers of repeated trials on the robustness of results is not explored, which is essential for guiding future experimental designs.

(4) Similarly, discussion on the minimal number of trials or stimulus conditions required for reliable generalization is lacking. Clearly outlining these basic experimental requirements would be highly beneficial for future users of this method.

Reviewer #3: This paper describes a technique that estimates the temporal evolution of activity in the visual cortex. The technique uses fMRI to estimate visual field maps and then employs a forward model to predict the MEG response. This prediction is done across time, such that each field map has an estimated temporal response accuracy for predicting the MEG response.

Overall assessment:

This paper provides a powerful tool to estimate of the contribution of each field map to visual responses over time. The technical aspects are solid, rigorous cross-validation is used to show that the model can explain responses to left out stimuli and the paper is clearly written. To further strengthen the paper, it would be particularly helpful to show the degree to which the proposed approach can replicate known temporal dynamics of the visual system. Moreover, there are some methodological and conceptual issues that need to be addressed.

Major comments:

1) Can the proposed method reproduce known temporal dynamics in the visual system? Previous intracranial studies measured onset latencies in the visual stream in humans (e.g. Martin et al., 2019, JNeurosci, Temporal Dynamics and Response Modulation across the Human Visual System in a Spatial Attention Task: An ECoG Study and Groen et al., 2022, JNeurosci, Temporal Dynamics of Neural Responses in Human Visual Cortex). Both these studies found that IPS has a particularly early response onset. Can the proposed model reproduce this finding?

2) What could be an explanation be for the delayed responses in V2 compared to V3 in Figure 3A and 3B?

3) The results and conclusion confuse measured responses and estimates of contributions. The contributions were not directly measured. (e.g. first sentence of the results: “To measure the time-courses of visual field maps...” and “We non-invasively measured the contribution…” )

4) Several studies have shown that evoked potentials, as measured here with MEG, are not directly related to the BOLD response measured with fMRI. In visual cortex, for example, it has been shown that these two measurements integrate differently across space (e.g. Winawer et al., 2013, Current Biology, Asynchronous Broadband Signals Are the Principal Source of the BOLD Response in Human Visual Cortex), with different non-linearities. Other studies comparing MEG and fMRI further showed that different neural response types result in different predicted spatial distributions (e.g. Kupers et al., 2021, A visual encoding model links magnetoencephalography signals to neural synchrony in human cortex). It should be discussed how such different differences in signals affect/are accounted for in the proposed method.

5) More background should be provided for testing and training across the magnetometers and planar gradiometers, as it is unclear to the reader whether these different sensors may emphasize different types of neural responses and whether these measurements would be expected to match or mismatch?

Minor:

The methods should state which degree of visual angle was spanned by the stimuli (assuming 5.34 degrees diameter based on Figure 5?).

It is unclear in the Figure 1 caption that r = 0.9-1.0 is meant to indicates a min-max range.

In the introduction “As a true model of neuronal activity” the term ‘true’ seems overstated.

In the method sentence: “Before the data matrices were fed into the ridge regression, the datapoints across all sensor and stimuli” sensor should be plural.

**********

**Have the authors made all data and (if applicable) computational code underlying the findings in their manuscript fully available?**

The PLOS Data policy requires authors to make all data and code underlying the findings described in their manuscript fully available without restriction, with rare exception (please refer to the Data Availability Statement in the manuscript PDF file). The data and code should be provided as part of the manuscript or its supporting information, or deposited to a public repository. For example, in addition to summary statistics, the data points behind means, medians and variance measures should be available. If there are restrictions on publicly sharing data or code —e.g. participant privacy or use of data from a third party—those must be specified.

Reviewer #1: **No:**  The data and code were not available for review. The data availability statement said "Analysis code will be made publicly available on GitHub. Minimally preprocessed data will be made available upon request, due to The Netherlands and EU General Data Protection Regulation (GDPR) compliance."

Reviewer #2: **No:**

Reviewer #3: **No:**  The authors state that Minimally preprocessed data will be made available upon request, due to The Netherlands and EU General Data Protection Regulation (GDPR) compliance.

**********

PLOS authors have the option to publish the peer review history of their article (what does this mean?). If published, this will include your full peer review and any attached files.

If you choose “no”, your identity will remain anonymous but your review may still be made public.

**Do you want your identity to be public for this peer review?**  For information about this choice, including consent withdrawal, please see our Privacy Policy.

Reviewer #1: **Yes:**  Laetitia Grabot

Reviewer #2: **Yes:**  Zitong Lu

Reviewer #3: No

[NOTE: If reviewer comments were submitted as an attachment file, they will be attached to this email and accessible via the submission site. Please log into your account, locate the manuscript record, and check for the action link "View Attachments". If this link does not appear, there are no attachment files.]

**Figure resubmission:**

While revising your submission, please upload your figure files to the Preflight Analysis and Conversion Engine (PACE) digital diagnostic tool, https://pacev2.apexcovantage.com/. PACE helps ensure that figures meet PLOS requirements. To use PACE, you must first register as a user. Registration is free. Then, login and navigate to the UPLOAD tab, where you will find detailed instructions on how to use the tool. If you encounter any issues or have any questions when using PACE, please email PLOS at figures@plos.org. Please note that Supporting Information files do not need this step. If there are other versions of figure files still present in your submission file inventory at resubmission, please replace them with the PACE-processed versions.

**Reproducibility:**

To enhance the reproducibility of your results, we recommend that authors of applicable studies deposit laboratory protocols in protocols.io, where a protocol can be assigned its own identifier (DOI) such that it can be cited independently in the future. Additionally, PLOS ONE offers an option to publish peer-reviewed clinical study protocols. Read more information on sharing protocols at https://plos.org/protocols?utm_medium=editorial-email&utm_source=authorletters&utm_campaign=protocols

# Author comment: Author response to Decision Letter 1

**DOI:** 10.1371/journal.pcbi.1014434.r002

4 Feb 2026

Attachment Submitted filename: Response to Reviewers.pdf

# Peer review: Decision Letter 1

**DOI:** 10.1371/journal.pcbi.1014434.r003

**Authors:** Hugues Berry, Yuanning Li, Hugues Berry, Yuanning Li

17 Mar 2026

PCOMPBIOL-D-25-01036R1

Non-invasive mapping of the temporal processing hierarchy in the human visual cortex

PLOS Computational Biology

Dear Dr. Eickhoff,

Thank you for submitting your manuscript to PLOS Computational Biology. After careful consideration, we feel that it has merit but does not fully meet PLOS Computational Biology's publication criteria as it currently stands. Therefore, we invite you to submit a revised version of the manuscript that addresses the points raised during the review process.

Please submit your revised manuscript by May 17 2026 11:59PM. If you will need more time than this to complete your revisions, please reply to this message or contact the journal office at ploscompbiol@plos.org. When you're ready to submit your revision, log on to https://www.editorialmanager.com/pcompbiol/ and select the 'Submissions Needing Revision' folder to locate your manuscript file.

Please include the following items when submitting your revised manuscript:

* A letter that responds to each point raised by the editor and reviewer(s). You should upload this letter as a separate file labeled 'Response to Reviewers'. This file does not need to include responses to formatting updates and technical items listed in the 'Journal Requirements' section below.

* A marked-up copy of your manuscript that highlights changes made to the original version. You should upload this as a separate file labeled 'Revised Manuscript with Track Changes'.

* An unmarked version of your revised paper without tracked changes. You should upload this as a separate file labeled 'Manuscript'.

If you would like to make changes to your financial disclosure, competing interests statement, or data availability statement, please make these updates within the submission form at the time of resubmission. Guidelines for resubmitting your figure files are available below the reviewer comments at the end of this letter

We look forward to receiving your revised manuscript.

Kind regards,

Yuanning Li

Academic Editor

PLOS Computational Biology

Hugues Berry

Section Editor

PLOS Computational Biology

**Reviewers' comments:**

Reviewer's Responses to Questions

**Comments to the Authors:**

**Please note here if the review is uploaded as an attachment.**

Reviewer #1: The authors have addressed most of my previous comments, but several important issues remain. Although they now provide solid statistical evidence showing that V1 responses occur earlier than those in other visual regions, the data do not fully support claims of a hierarchical progression across extrastriate areas, as latency increases along the visual stream were not directly tested. In addition, the use of tSSS preprocessing renders magnetometer and gradiometer signals non-independent, which limits the relevance of assessing model robustness across sensor types. The manuscript also does not provide guidance regarding the number of participants required to draw reliable conclusions, an aspect that would be particularly valuable in a methodological paper. Finally, the discussion still lacks a clear and specific articulation of the unique questions enabled by the proposed approach, a point previously raised by all reviewers.

Major points:

1. The preprocessing of the MEG data should be described in sufficient detail in the present manuscript so that readers do not need to refer to the first article using this dataset. It appears that temporal Signal Space Separation (tSSS) was applied, as is common in MEG preprocessing. However, this procedure introduces signal mixing between magnetometers and gradiometers (e.g., Garcés et al., 2017, Sensors, Choice of Magnetometers and Gradiometers after Signal Space Separation). As a result, assessing the robustness of the model across sensor types is of limited interest, since the signals are no longer independent. This point should at least be discussed.

2. The authors only tested whether the latency in extrastriate regions are different from V1 latency, but they did not test whether there was an increase in latency along visual regions. They cannot claim to report “a processing hierarchy across extrastriate visual fields maps” (p2 l45).

3. The authors claim that their method enables the identification of a temporal hierarchy in visual processing. However, when Reviewer 3 requested clarification regarding the observed delay between V2 and V3 responses or asked whether a known finding in the literature might be replicated (early IPS onset), the authors argued that the sample size was too small to draw firm conclusions. This raises an important question: how many participants would be required to reliably detect such differences? Providing at least an estimate or power analysis would be particularly valuable in a methodological paper, as it would help guide future users regarding the expected sensitivity and practical requirements of the approach.

4. Finally, in line with the two other reviewers’ comments, I agree that the manuscript would benefit from a more explicit discussion of how the proposed method enables the field to address specific questions that could not be answered with previous approaches. Clearly articulating these added-value aspects would substantially strengthen the paper, as the current discussion remains too general.

Minor points:

- One of my previous points has not been fully addressed. I had asked for a figure showing the predicted sensor-level activity associated with the model’s predicted responses, so that readers can directly appreciate what these predictions look like. The authors indicate that such a plot is available in their previous article (Eickhoff et al., 2024, Imaging Neuroscience). However, that article does not include predictions derived from visual field maps, which constitute the novelty of the present study.

I still believe it would be highly informative to visualize these predictions within a classical ERP framework, as this would allow readers to assess which components align more closely with the different visual field map–based predictions.

- One limitation of the model that would be worth discussing is that it only considers static stimuli (as in the prediction stage, a single snapshot of the presented stimuli is used). How could you adapt your model to dynamically evolving stimuli (for instance a moving bar)?

- In Figure 3C, it is not possible to clearly distinguish which violin plots are shown as opaque or transparent (indicating whether latencies differ significantly from V1).

Reviewer #2: Thanks for the revision. I am good with this version.

Reviewer #3: The authors have addressed all my comments.

**********

**Have the authors made all data and (if applicable) computational code underlying the findings in their manuscript fully available?**

The PLOS Data policy requires authors to make all data and code underlying the findings described in their manuscript fully available without restriction, with rare exception (please refer to the Data Availability Statement in the manuscript PDF file). The data and code should be provided as part of the manuscript or its supporting information, or deposited to a public repository. For example, in addition to summary statistics, the data points behind means, medians and variance measures should be available. If there are restrictions on publicly sharing data or code —e.g. participant privacy or use of data from a third party—those must be specified.

Reviewer #1: **No:**  Code is available on github with example data for 1 participant.

Reviewer #2: Yes

Reviewer #3: **No:**  Will be provided after acceptance: The minimal anonymized data necessary to replicate these study findings have been deposited in [OpenNeuro full link to be provided after acceptance]

**********

PLOS authors have the option to publish the peer review history of their article (what does this mean?). If published, this will include your full peer review and any attached files.

If you choose “no”, your identity will remain anonymous but your review may still be made public.

**Do you want your identity to be public for this peer review?**  For information about this choice, including consent withdrawal, please see our Privacy Policy.

Reviewer #1: **Yes:**  Laetitia Grabot

Reviewer #2: **Yes:**  Zitong Lu

Reviewer #3: No

[NOTE: If reviewer comments were submitted as an attachment file, they will be attached to this email and accessible via the submission site. Please log into your account, locate the manuscript record, and check for the action link "View Attachments". If this link does not appear, there are no attachment files.]

**Figure resubmission:**

While revising your submission, we strongly recommend that you use PLOS’s NAAS tool (https://ngplosjournals.pagemajik.ai/artanalysis) to test your figure files. NAAS can convert your figure files to the TIFF file type and meet basic requirements (such as print size, resolution), or provide you with a report on issues that do not meet our requirements and that NAAS cannot fix.

After uploading your figures to PLOS’s NAAS tool - https://ngplosjournals.pagemajik.ai/artanalysis, NAAS will process the files provided and display the results in the "Uploaded Files" section of the page as the processing is complete. If the uploaded figures meet our requirements (or NAAS is able to fix the files to meet our requirements), the figure will be marked as "fixed" above. If NAAS is unable to fix the files, a red "failed" label will appear above. When NAAS has confirmed that the figure files meet our requirements, please download the file via the download option, and include these NAAS processed figure files when submitting your revised manuscript.

**Reproducibility:**

To enhance the reproducibility of your results, we recommend that authors of applicable studies deposit laboratory protocols in protocols.io, where a protocol can be assigned its own identifier (DOI) such that it can be cited independently in the future. Additionally, PLOS ONE offers an option to publish peer-reviewed clinical study protocols. Read more information on sharing protocols at https://plos.org/protocols?utm_medium=editorial-email&utm_source=authorletters&utm_campaign=protocols

# Author comment: Author response to Decision Letter 2

**DOI:** 10.1371/journal.pcbi.1014434.r004

31 May 2026

Attachment Submitted filename: Response_to_Reviewers_auresp_2.pdf

# Peer review: Decision Letter 2

**DOI:** 10.1371/journal.pcbi.1014434.r005

**Authors:** Hugues Berry, Yuanning Li, Hugues Berry, Yuanning Li, Hugues Berry, Yuanning Li

11 Jun 2026

Dear Eickhoff,

We are pleased to inform you that your manuscript 'Non-invasive mapping of the temporal processing hierarchy in the human visual cortex' has been provisionally accepted for publication in PLOS Computational Biology.

Before your manuscript can be formally accepted you will need to complete some formatting changes, which you will receive in a follow up email. A member of our team will be in touch with a set of requests.

Please note that your manuscript will not be scheduled for publication until you have made the required changes, so a swift response is appreciated.

IMPORTANT: The editorial review process is now complete. PLOS will only permit corrections to spelling, formatting or significant scientific errors from this point onwards. Requests for major changes, or any which affect the scientific understanding of your work, will cause delays to the publication date of your manuscript.

Should you, your institution's press office or the journal office choose to press release your paper, you will automatically be opted out of early publication. We ask that you notify us now if you or your institution is planning to press release the article. All press must be co-ordinated with PLOS.

Thank you again for supporting Open Access publishing; we are looking forward to publishing your work in PLOS Computational Biology.

Best regards,

Yuanning Li

Academic Editor

PLOS Computational Biology

Hugues Berry

Section Editor

PLOS Computational Biology

***********************************************************

Reviewer's Responses to Questions

**Comments to the Authors:**

**Please note here if the review is uploaded as an attachment.**

Reviewer #1: The authors have addressed all my comments.

**********

**Have the authors made all data and (if applicable) computational code underlying the findings in their manuscript fully available?**

The PLOS Data policy requires authors to make all data and code underlying the findings described in their manuscript fully available without restriction, with rare exception (please refer to the Data Availability Statement in the manuscript PDF file). The data and code should be provided as part of the manuscript or its supporting information, or deposited to a public repository. For example, in addition to summary statistics, the data points behind means, medians and variance measures should be available. If there are restrictions on publicly sharing data or code —e.g. participant privacy or use of data from a third party—those must be specified.

Reviewer #1: Yes

**********

PLOS authors have the option to publish the peer review history of their article (what does this mean?). If published, this will include your full peer review and any attached files.

If you choose “no”, your identity will remain anonymous but your review may still be made public.

**Do you want your identity to be public for this peer review?**  For information about this choice, including consent withdrawal, please see our Privacy Policy.

Reviewer #1: **Yes:**  Laetitia Grabot

# Editor report: Acceptance letter

**DOI:** 10.1371/journal.pcbi.1014434.r006

**Authors:** Hugues Berry, Yuanning Li, Hugues Berry, Yuanning Li, Hugues Berry, Yuanning Li

PCOMPBIOL-D-25-01036R2

Non-invasive mapping of the temporal processing hierarchy in the human visual cortex

Dear Dr Eickhoff,

I am pleased to inform you that your manuscript has been formally accepted for publication in PLOS Computational Biology. Your manuscript is now with our production department and you will be notified of the publication date in due course.

The corresponding author will soon be receiving a typeset proof for review, to ensure errors have not been introduced during production. Please review the PDF proof of your manuscript carefully, as this is the last chance to correct any errors. Please note that major changes, or those which affect the scientific understanding of the work, will likely cause delays to the publication date of your manuscript.

Soon after your final files are uploaded, unless you have opted out, the early version of your manuscript will be published online. The date of the early version will be your article's publication date. The final article will be published to the same URL, and all versions of the paper will be accessible to readers.

For Research, Software, and Methods articles, you will receive an invoice from PLOS for your publication fee after your manuscript has reached the completed accept phase. If you receive an email requesting payment before acceptance or for any other service, this may be a phishing scheme. Learn how to identify phishing emails and protect your accounts at https://explore.plos.org/phishing.

Thank you again for supporting PLOS Computational Biology and open-access publishing. We are looking forward to publishing your work!

With kind regards,

Anitha Samidurai

PLOS Computational Biology | Carlyle House, Carlyle Road, Cambridge CB4 3DN | United Kingdom ploscompbiol@plos.org | Phone +44 (0) 1223-442824 | ploscompbiol.org | @PLOSCompBiol
